# New Cation Sensors Based on Eugenol-Derived Azo Dyes

**DOI:** 10.3390/molecules30132788

**Published:** 2025-06-28

**Authors:** José R. A. Coelho, Ana Rita F. Pacheco, Diogo C. Domingues, Ana Rita O. Rodrigues, Akani A. Temitope, Paulo J. G. Coutinho, Maria José G. Fernandes, Elisabete M. S. Castanheira, M. Sameiro T. Gonçalves

**Affiliations:** 1 Chemistry Centre of the University of Minho (CQ-UM), Department of Chemistry, University of Minho, Campus de Gualtar, 4710-057 Braga, Portugal; a85799@alumni.uminho.pt (J.R.A.C.); diogodomingues.2408@gmail.com (D.C.D.); akanniabdulakeemt@gmail.com (A.A.T.); mjfernandes@quimica.uminho.pt (M.J.G.F.); 2 Physics Centre of Minho and Porto Universities (CF-UM-UP), Department of Physics, University of Minho, Campus de Gualtar, 4710-057 Braga, Portugalritarodrigues@fisica.uminho.pt (A.R.O.R.); pcoutinho@fisica.uminho.pt (P.J.G.C.); ecoutinho@fisica.uminho.pt (E.M.S.C.); 3 Associate Laboratory LaPMET, University of Minho, Campus de Gualtar, 4710-057 Braga, Portugal

**Keywords:** eugenol, phenolic compounds, azo dyes, cation detection, colorimetric sensors

## Abstract

Eugenol-based azo dyes illustrate how bio-sourced compounds like eugenol can be transformed through synthetic processes into functional and colorful compounds. The main purpose of the present work was to develop new responsive colorimetric sensors for metal cations based on eugenol-derived azo compounds. The incorporation of the azo group into the eugenol framework allows for strong electronic interactions with metal cations, leading to distinct color changes observable to the naked eye. These azo-eugenol dyes exhibit shifts in their UV-Vis absorption spectra upon complexation with metal cations such as copper (Cu^2+^) and lead (Pb^2+^), making them effective sensors for environmental and analytical applications. The eugenol-based azo dyes were subjected to photophysical studies to understand selectivity, response time, and stability in relation to metal cations, which will be a starting point for the monitoring of toxic metal contaminants in aqueous environments.

## 1. Introduction

Azo dyes, known for their diversity of appealing colors and stability, have been widely used in various applications such as textiles, food, and analytical chemistry, due to their ease of synthesis, structural variability, and unique chromogenic tonalities to the naked eye, where reds, oranges, and yellows predominate [1,2,3,4,5,6]. The exact coloration of the respective dyes is based on the existence of a chromophore group (-N=N-), associated with the nature and position of the substituents connected to the aromatic systems, where the amplification of the degree of conjugation of the π system enhances the absorption of light at longer wavelengths [7,8]. These dyes can undergo significant color changes in response to the binding of specific cations, enabling rapid, visual detection of metal ions [9,10,11]. Among these, azo dyes derived from natural sources, like eugenol, may offer a promising eco-friendly alternative to full synthetic dyes, where it is possible to combine the physicochemical and biological properties inherent in phenolic compounds, known to have significant antioxidant activities [12,13].

Eugenol, the major secondary metabolite found in clove oil (*Syzygium aromaticum*) and present in other essential oils, provides a versatile basis for the synthesis of azo dyes due to its reactive functional groups and aromatic structure. Azo dyes synthesized from eugenol are not only potential environmentally benign, but also possess unique photophysical properties, making them ideal candidates for colorimetric sensing applications [14,15,16]. From a structural perspective, eugenol is a phenolic compound, meaning that the existence of a hydroxyl group makes it a suitable ligand for complexing with metals. Another crucial aspect is the role of the methoxyl group in the orientation of the ligand in relation to the metal center, which positions the donor atom in the metal’s coordination sphere [17]. As a ligand, eugenol can form complexes with important biometals such as Fe^2+^ and Fe^3+^ cations. The eugenol moiety has been already reported as a colorimetric probe for iron(III), where it displays complexes that have a blue-green color (λ_max_ = 653 nm) [18]. Thus, eugenol-derived azo dyes present a novel, sustainable, and effective approach for developing selective and sensitive colorimetric sensors for cation detection.

This feature is particularly valuable in environmental monitoring, medical diagnostics, and industrial processes, where detecting toxic or essential cations with high sensitivity and selectivity is crucial. Metals present a dichotomy based on their dose and their biological importance [19]. Some metals are essential in metabolic processes, where excess or deficiency can lead to severe damage. Other metals are real poisons that tend to bioaccumulate in the environment. Copper is a biologically relevant element; however, prominent levels of Cu^2+^ can contribute to the development of neurodegenerative diseases (such as Alzheimer’s disease, Menkes’ syndrome, or Wilson’s disease), prion disease, hypoglycemia, and dyslexia, and also to the progression of cancer [20,21,22]. On the other hand, lead (Pb^2+^) is described as a poison that can affect many organs in the human body, damaging the brain, nervous system, blood, digestive system, and also the reproductive system, due to its ability to mimic and, in some cases, inhibit the action of calcium as a regulator of cellular function [23,24].

In recent years, scientific research efforts have been directed toward significant advances in the detection of various metal cations, particularly through the development of chemosensors for Cu^2+^ and Pb^2+^ ions, which have recently been described in detail [25,26]. Organic colorimetric chemical sensors, such as thioureas, Schiff bases (imines), pyridines, thiophenes, thiazoles, indoles, calixarenes, porphyrins, crown ethers, and BODIPYs, are promising candidates [27,28,29,30,31]. Furthermore, the valorization of natural products has been an emerging field in the design of new ligands for the detection of metal cations.

Taking these facts into consideration, the present work focuses on broadening the scope of application of eugenol-based azo dyes—including previously reported structures developed for other purposes and applications [14,16]—by evaluating their use in the detection of various metal cations, with promising results in the colorimetric sensing of lead(II) and copper(II) ions.

## 2. Results and Discussion

### 2.1. Synthesis of Eugenol Azo Dyes ***3a***–***e***

**Scheme 1:**
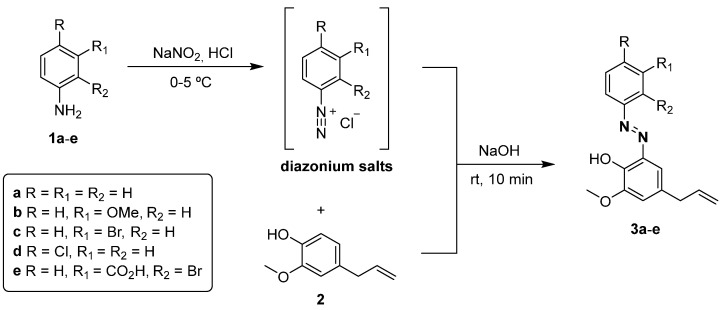
Synthesis of eugenol azo dyes **3a**–**e**.

Azo dyes incorporating eugenol (4-allyl-2-methoxyphenol) into their structures have been synthesized following a conventional methodology. In this process, eugenol was coupled with diazonium salts of aniline-based amines to form the azo compounds. Aniline **1a**, 3-methoxyaniline **1b**, 3-bromoaniline **1c**, 4-chloroaniline **1d**, and 3-amino-2-bromobenzoic acid **1e** were reacted in an acidic medium with sodium nitrite, at low temperature, producing diazonium salts, which were added to eugenol **2** in a basic medium, to yield 4-allyl-2-methoxy-6-(phenyldiazenyl)phenol **3a** [14], 4-allyl-2-methoxy-6-((3-methoxyphenyl)diazenyl)phe-nol **3b** [16], 4-allyl-2-((3-bromophenyl)diazenyl)-6-methoxyphenol **3c**, 4-allyl-2-((4-chlorophenyl)diazenyl)-6-methoxy-phenol **3d** [14], and 3-((5-allyl-2-hydroxy-3-methoxyphenyl)diazenyl)-2-bromobenzoic acid **3e** (Scheme 1).

Compounds **3a**–**e** were obtained as orange and red solids in moderate yields (14–44%). These yields result due to the fact that eugenol has very limited solubility in water, meaning that the reaction tends not to be complete and requires purification of the crude product obtained using chromatographic techniques, which also leads to significant losses. Considering the electronic nature of the substituent groups of eugenol, as well as the possible steric impediments associated with the allyl side chain, the site for azo-functionalization will occur in the *ortho* position relative to the hydroxyl group. The respective compounds were fully characterized by using the usual analytical techniques: ^1^H NMR, ^13^C NMR, FTIR spectroscopy, and HRMS (see Figures S1–S22, Supplementary Materials).

In the ^1^H NMR spectra, it is possible to distinguish different signals for aliphatic protons, namely, methylenic (*δ* 3.37–3.43 ppm) and methoxyl (*δ* 3.84–3.95 ppm) groups, in addition to the expected protons of the eugenol’s double bond as multiplets CH_2_ (*δ* 5.06–5.19 ppm) and CH (*δ* 5.96–6.07 ppm). With the occupation of the most activated position for the occurrence of azo coupling, the respective aromatic signals of eugenol (H-3 and H-4 or H-4 and H-6, in the case of **3e**) will correspond to two doublets (*J* 1.6 or 2.0 Hz) or be included in a multiplet (H-5 in **2b**) (*δ* 6.80–7.47 ppm). For amines, the aromatic protons will appear at specific chemical shifts, depending on the electronic nature and position of the substituents present.

The ^13^C NMR spectra of all compounds showed signals of the aliphatic carbons from the CH_2_ (δ 38.81–39.55 ppm) and OCH_3_ groups (*δ* 56.14–56.48 ppm), in addition to carbons of the eugenol’s double bond CH_2_ (*δ* 116.03–116.28 ppm) and CH (*δ* 136.96–137.42 ppm). The carbons of the aromatic rings were also shown (*δ* 112.15–160.38 ppm). For compound **3e**, it is possible to assign the carbon in the carbonyl group of the carboxylic acid function at *δ* 168.04 ppm. The FTIR spectra also confirmed the presence of a CO_2_H group for compound **3e**, showing the corresponding C=O stretching vibration bands at 1695 cm^−1^.

### 2.2. UV/Vis Absorption of the Eugenol Azo Dyes ***3a***–***e***

**Figure 1:**
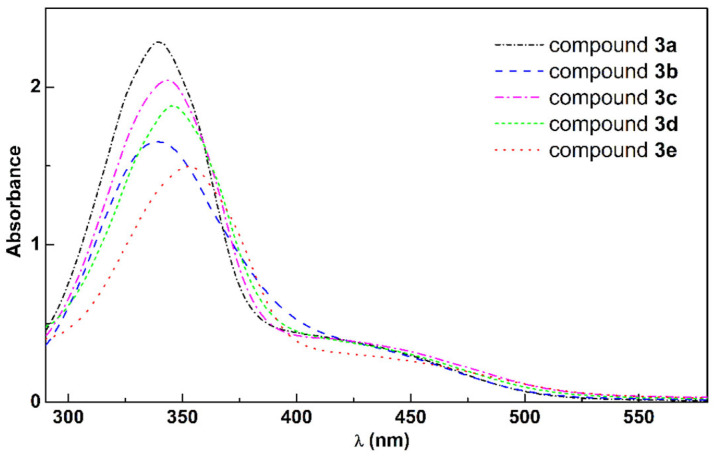
Absorption spectra of compounds **3a**–**e** in neutral ethanolic solution (2 × 10^−5^ M).

The eugenol derivatives **3a**–**e** exhibit absorption in the UV/visible region, and their absorption spectra are illustrated in Figure 1, showing an intense absorption band in the UV range, at around 345 nm, and another band in the blue region, justifying the yellowish color. The strong red shift of the UV band relative to the eugenol UV/Vis absorption spectrum (maximum at ~280 nm) [32] points to an extended conjugation between the aromatic moieties, including the azo group. The band in the visible region is probably due to an *n*→π* transition involving N atoms.

The compounds were compared, and small variations are detected in the UV band position and intensity, reflecting the influence of the substituents in the phenyl ring linked to the azo moiety. Maximum absorption wavelengths (λ_max_) and molar absorption coefficients (ε) are indicated in Table 1. Compound **3a**, with no substituents in the phenyl ring, display the highest absorption coefficient. Compound **3e**, possessing two substituents in this ring (–Br and –COOH groups in the *ortho* position relative to each other), presents a lower absorption coefficient and a significant bathochromic shift, probably due to the extension of the conjugation of the aromatic ring to the carbonyl group of –COOH.

**Figure 2:**
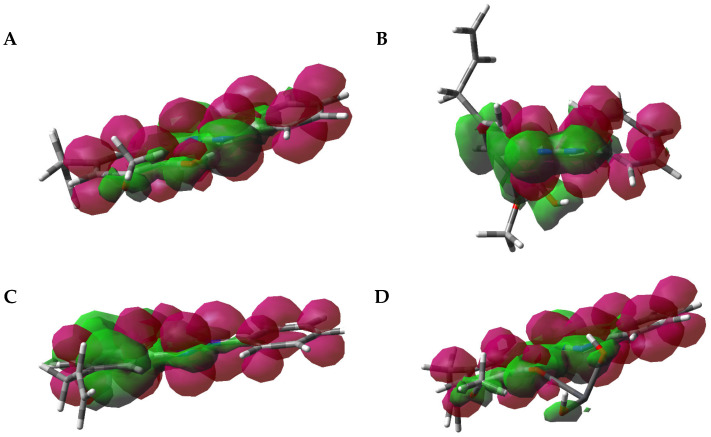
Ground state equilibrium geometries and electron density variation upon excitation to the first excited state of *trans* (**A**) and *cis* (**B**) conformers, the hydrazone tautomer (**C**), and the Pb^2+^ complex (**D**). The green and purple areas correspond to decreases and increases in electron density, respectively.

Ground state equilibrium geometries for *cis* and *trans* conformers of compound **3a**, as well as its hydrazone tautomer, are shown in Figure 2, together with electron density variation upon excitation to the first excited state (Cartesian coordinates in Table S4 of Supplementary Materials). Vibrational analysis confirmed, through the absence of imaginary frequencies, that no metastable molecular geometries were obtained; this allowed us to place the free energies of the *cis* conformer and the hydrazone tautomer, respectively, at 19.5 kcal/mol and 1.83 kcal/mol above the *trans* form of compound **3a**. This result predicts that 4.4% of compound **3a** exists in its hydrazone form at room temperature (25 °C).

Table 2 shows the obtained transition wavelengths and oscillator strengths for the first eight excited states for both conformers and for the hydrazone tautomer. Considering the low oscillator strength for the first excited state and the electron density variations in Figure 2, it is possible to conclude that, in both *cis* and *trans* conformers, the first electronic transition is mainly of the *n*→π* type, as a decrease occurs along the azo backbone and an increase occurs in the π electron system above the molecular plane. The *cis* conformer shows higher oscillator strength as non-planarity results in a better overlap between *n* and π* molecular orbitals. The hydrazone tautomer, which is planar like the azo tautomer, clearly shows a π→π* transition, corresponding to a higher oscillator strength. Comparing the experimental spectrum (Figure 1) in the region above 440 nm with the oscillator strengths in Table 2 (7.4 × 10^−4^ for the *trans* conformer vs. 0.049 and 0.13 for the tautomer), it is possible to conclude that the absorption spectrum mainly corresponds to the *trans* conformer, as expected from its lower free energy.

**Figure 3:**
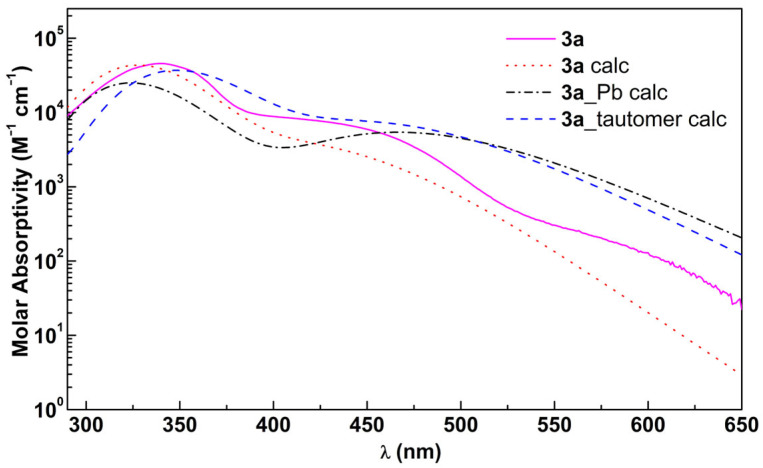
Calculated absorption spectra (in log scale) of compound **3a** (**3a** calc), its hydrazone tautomer (**3a**_tautomer calc), and its complex with Pb^2+^ (**3a**_Pb calc).

Prediction of the absorption spectral shape requires vibrational analysis of both ground and excited states in their equilibrium geometries. The geometry optimization of the first excited state resulted in a very high geometry variation, so that the S_0_ → S_1_ energy difference became very small (~1 eV). Thus, a simplified method was used, in which each electronic transition is considered to be a Gaussian function in wavenumber, with a fixed standard deviation corresponding to 0.4 eV (3226 cm^−1^). In Figure 3, a reasonable accordance is shown between the predicted spectrum and the experimental one, although the molar absorptivity of the S_0_ → S_2_ band is lower than the experimental value. The higher absorptivity in that region could be due to the contribution of the hydrazone tautomer that has a significant molar absorptivity in that spectral region.

### 2.3. Colorimetric Assays of Compounds ***3a***–***e*** as Ion Sensors

**Figure 4:**
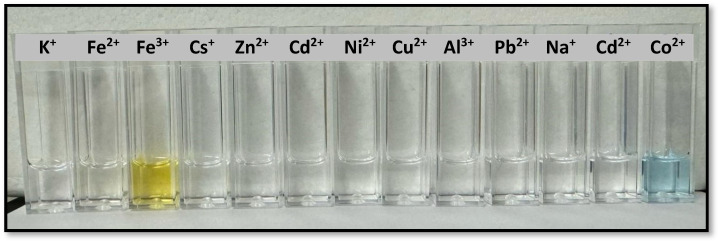
Solutions of the salts of several cations. Cd^2+^ was tested as a perchlorate salt (**left**) and a nitrate salt (**right**).

**Figure 5:**
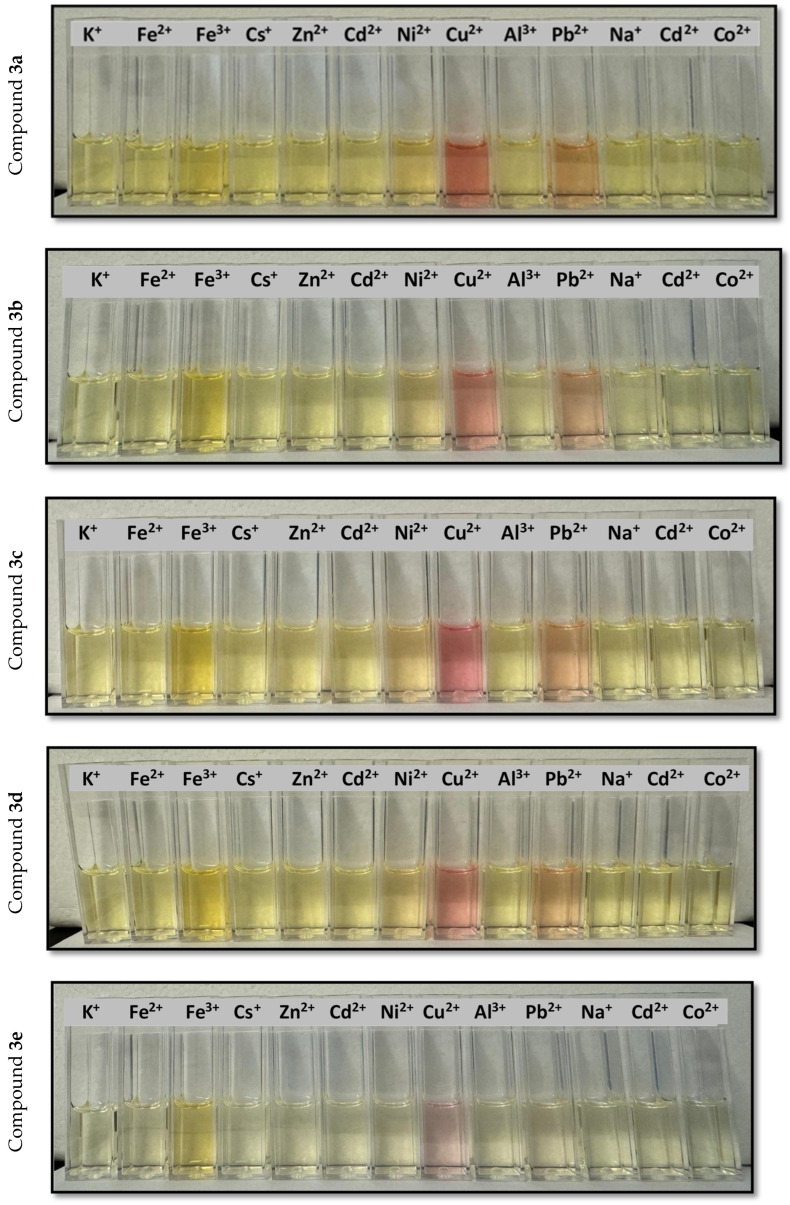
Solutions of compounds **3a**–**e** after the addition of 15 equiv. of the salts. Cd^2+^ was tested as a perchlorate salt (left) and a nitrate salt (right), and no differences were obtained.

The compounds **3a**–**e** were preliminary tested as sensors for several cations in order to obtain a colorimetric assay, which is especially useful for detecting pollutants and poisoning metals. The cations (15 equiv.) were added to compounds’ solutions, and the colors were compared between the neat cation solutions and the compound/cation solutions (Figure 4 and Figure 5).

Considering the objective of obtaining a colorimetric assay for the detection of cations, we find, in Figure 5, evidence that the compounds can be useful for the detection of copper(II) and lead(II), which display a clear change in color, despite compound **3e** seeming not useful for this determination.

Particularly, compound **3c** seems to be the most useful for Cu^2+^, demonstrating a clearly distinguishable and intense pink color in the presence of this cation. This color change can be observed at concentrations as low as 10 µM of cations, while for the other compounds, higher cation concentrations are needed. Here, the possibility of the detection of Pb^2+^ is especially valuable, because lead is described as a poison that can affect a large number of organs in human body. Therefore, the five compounds’ solutions were titrated with lead(II) cations.

### 2.4. Titration of Compound ***3c*** with Cu^2+^

**Figure 6:**
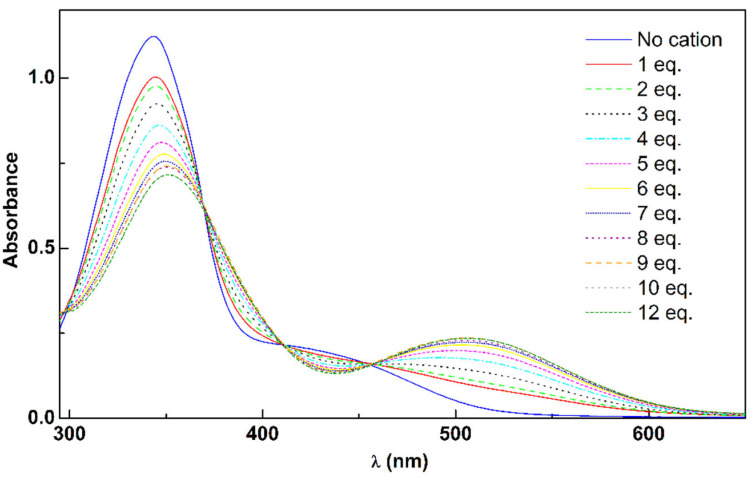
Absorption spectra of compound **3c** (1 × 10^−5^ M) with the addition of Cu^2+^. The spectra of 10 and 12 equiv. of Cu^2+^ are superposed.

Spectrophotometric titrations in ethanol were performed to further investigate the ability of compound **3c** to detect copper(II) ion. Therefore, increasing amounts of the cation were added to the compound solution, and the corresponding absorption spectra were obtained. A new absorption band, centered at around 530 nm, is observed (Figure 6), the maximum absorption of this band being attained at 10 equivalents of the cation, pointing to a high sensitivity. The presence of three clear isosbestic points indicates a complex interaction between the compound and Cu^2+^.

**Figure 7:**
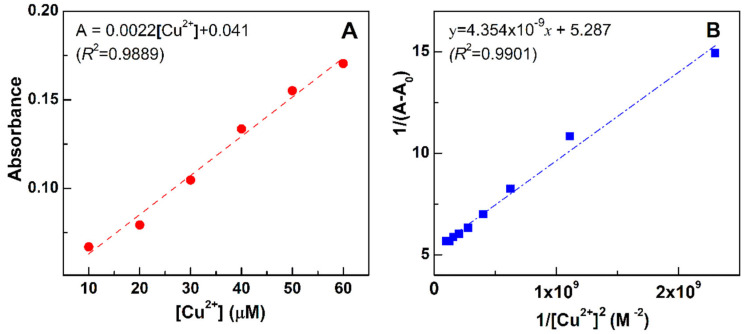
(**A**) Linear plot of absorbance at 540 nm versus Cu^2+^ concentration. (**B**) Benesi–Hildebrand plot for the spectrophotometric titration of compound **3c** with Cu^2+^ at 540 nm.

In these results, a linear relationship between the cation concentration and the absorbance of the new band at 540 nm (where the absorption of the neat compound is negligible) was observed up to 60 µM of cations (Figure 7A). From this plot, a limit of detection (LOD) as low as 0.16 µM was estimated. This value shows that compound **3c** can be very useful for the detection of Cu^2+^ in effluents and other samples, considering that the maximum level of copper in drinking water is 20.5 µM (WHO, 2008 [33]), well above the LOD and in the linear range of the calibration curve (Figure 7A). Moreover, the average concentration of copper in blood should not exceed 15.7–23.6 μM in normal conditions [34], also in the detectable range. This LOD value is lower than the ones reported for other sensors of copper(II) [35,36,37], making compound **3c** highly advantageous for this purpose.

To calculate the binding constant, the Benesi–Hildebrand equation was used, the *K_a_* value being determined from the ratio of the intercept to the slope of the Benesi–Hildebrand plot (Figure 7B). A linear relationship was obtained only for *n* = 2 (while for *n* = 1, the plot is not linear), indicating a binding stoichiometry of 1:2 between the compound and the copper (II) cation, with a corresponding binding constant of *K_a_* = 1.21 × 10^9^ M^−2^. Azo dyes based on eugenol (without the substituents R, R_1_, and R_2_) incorporated in silicone films have also shown the capability to detect Cu^2+^ metal ions, represented by a change in color of the film, similarly exhibiting a 1:2 stoichiometry [38].

### 2.5. Titration of Compounds ***3a***–***e*** with Pb^2+^

Considering the proof-of-concept obtained with copper(II) cations, having a low LOD value, we explored the possibility of the synthesized compounds acting as lead(II) sensors. For this purpose, each of the compounds **3a**–**e** was titrated with lead(II), taking into account the significant harmful environmental and health effects from high concentrations of lead [39]. Lead exposure can have serious consequences for health, especially with children. The WHO has established “International Lead Poisoning Prevention Week” (every year in October) to prevent childhood lead exposure and to stop the use of lead in paint [40].

**Figure 8:**
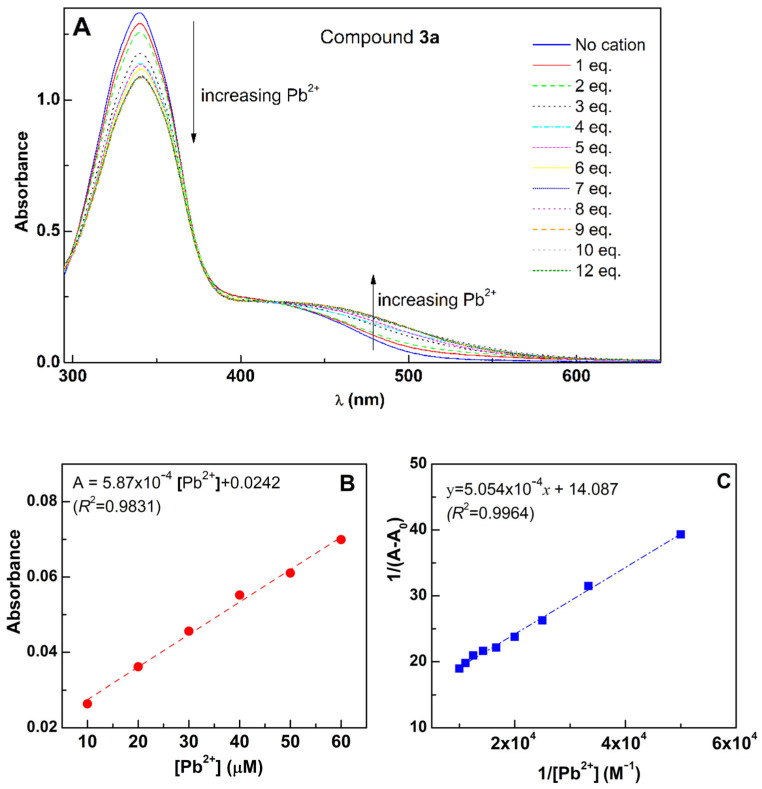
(**A**) Absorption spectra of compound **3a** (1 × 10^−5^ M) with the addition of Pb^2+^. (**B**) Linear plot of absorbance at 540 nm versus Pb^2+^ concentration. (**C**) Benesi–Hildebrand plot for the spectrophotometric titration of compound **3a** with Pb^2+^.

**Figure 9:**
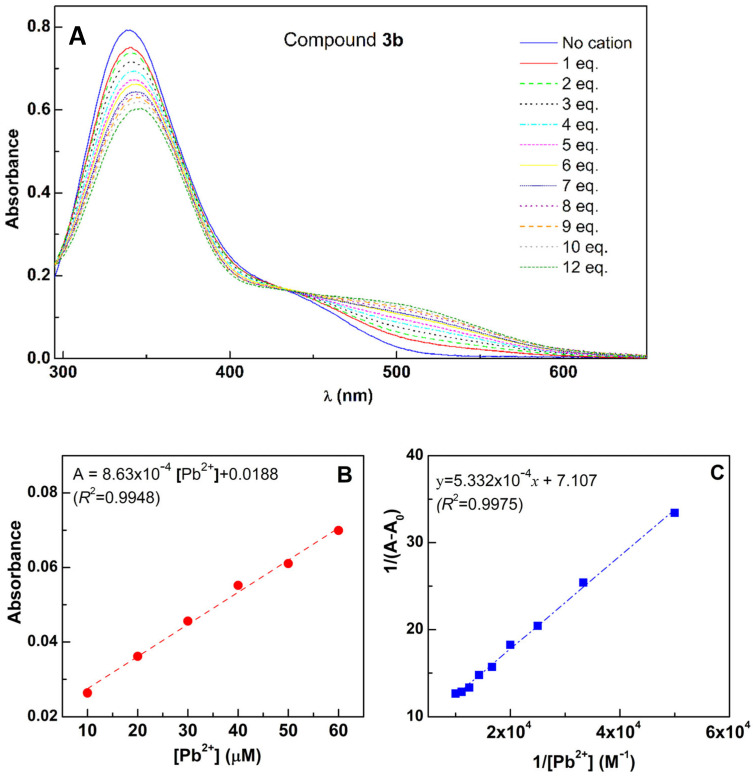
(**A**) Absorption spectra of compound **3b** (1 × 10^−5^ M) with the addition of Pb^2+^. (**B**) Linear plot of absorbance at 540 nm versus Pb^2+^ concentration. (**C**) Benesi–Hildebrand plot for the spectrophotometric titration of compound **3b** with Pb^2+^.

**Figure 10:**
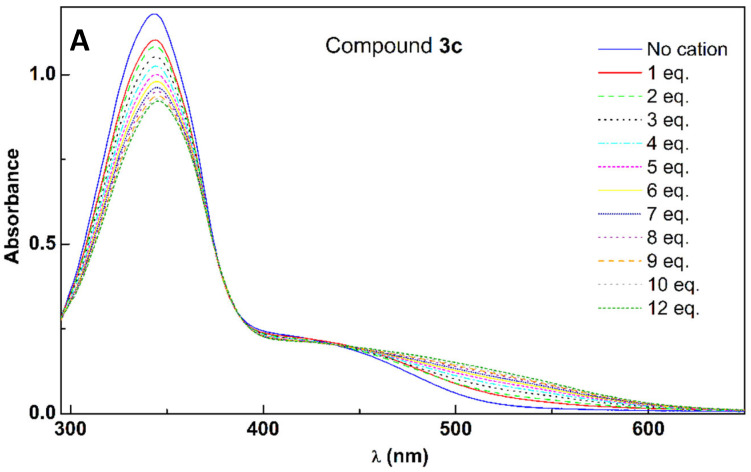

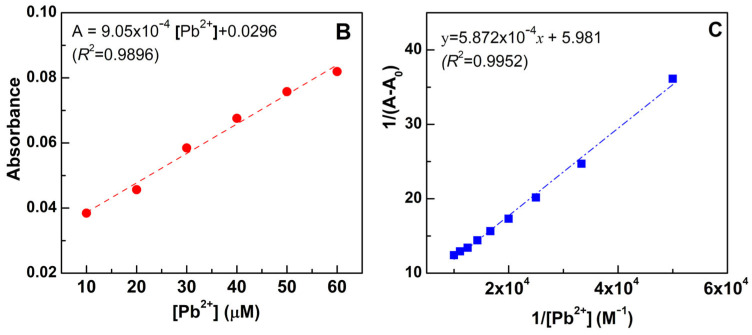
(**A**) Absorption spectra of compound **3c** (1 × 10^−5^ M) with the addition of Pb^2+^ (**B**) Linear plot of absorbance at 540 nm versus Pb^2+^ concentration. (**C**) Benesi–Hildebrand plot for the spectrophotometric titration of compound **3c** with Pb^2+^.

As predicted by the less prominent variation in color upon Pb^2+^ addition (comparing with copper), the spectral effects of the presence of the ion are much less pronounced, compound 3e not being sensitive to the presence of Pb^2+^ in the titration (as inferred from the color invariance in Figure 5). Moreover, the titration of compound 3d also shows very small spectral changes with the addition of lead cations. Therefore, the titration of compounds 3a, 3b, and 3c with Pb^2+^ were analyzed to determine the binding constants and LOD values (Figure 8, Figure 9 and Figure 10). The parameters for the interaction of each compound, 3a–c, with lead cations are summarized in Table 3. It must be pointed out that for compounds 3a, 3b, and 3c, the Benesi–Hildebrand plots (Figure 8C, Figure 9C and Figure 10C) only display a good correlation for *n* = 1, indicating a 1:1 stoichiometry for the interaction of lead(II) with the azo compounds. In fact, the spectra for the titration of compounds with lead(II) are completely different from that of compound 3c with copper(II), pointing to a different kind of interaction of the azo dyes with the two cations.

In order to understand the spectral changes upon interaction with Pb^2+^, ab initio calculations were performed for coupling of the metal ion to the OH group of compound **3a**, [Pb(OH)_2_**3a**]^−^. The results are included in Table 2 and in Figure 2 and Figure 3. From the calculated spectrum, a significant decrease in molar absorptivity near 340 nm and an increase in the 450–550 nm spectral region is detected. This feature is in accordance with the observed variations in absorbance of compound **3a** with varying amounts of Pb^2+^ cations.

The WHO published guidelines on clinical management exposure to lead (2021), recommending that, for individuals with a blood lead concentration equal to or higher than 5 µg/dL (0.24 µM), the source of lead exposure must be identified and actions immediately taken [41]. US CDC (Centers for Disease Control and Prevention) uses a blood lead reference value (BLRV) of 3.5 µg/dL (0.17 µM) for children [42]. Given the high level of lead poisoning, compounds **3a**–**c** are promising as colorimetric sensors for determination of lead(II), considering the detectable range and the low LOD values.

## 3. Materials and Methods

### 3.1. Chemicals and Reagents

Melting points were determined using a Stuart SMP3 apparatus (Bibby Scientific, Staffordshire ST15 0SA, UK). Thin-layer chromatography (TLC) was performed on 0.20 mm thick precoated silica gel plates (Macherey-Nagel, Düren 52355, Germany). Visualization of the spots was carried out both with the naked eye and under UV light at 365 nm using a CN-15 camera system (Vilber Lourmat, Marne-la-Vallée 77202, France). Column chromatography was conducted on silica gel 60 (particle size 0.035–0.070 mm, Acros Organics, Geel, Belgium).

### 3.2. Analytical Instruments

FTIR spectra were determined on a Spectrum Two FTIR PerkinElmer spectrophotometer (PerkinElmer Inc., Waltham, MA 01821, USA). UV-Vis-NIR absorption spectra (200–800 nm) were obtained using a Shimadzu UV-3600 Plus spectrophotometer (Shimadzu Europa GmbH, Duisburg 47269, Germany). NMR spectra were recorded on a Bruker Avance III 400 spectrometer (Billerica, MA 01821, USA) operating at 400 MHz for ^1^H and 100.6 MHz for ^13^C nuclei, using the solvent residual peak as an internal reference at 25 °C. Chemical shifts (δ) are reported in parts per million (ppm) relative to TMS (δ_H_ = 0 ppm), and coupling constants (J) are given in hertz (Hz). Signal assignments were made based on chemical shifts, multiplicities, and J values and further confirmed by spin decoupling (double-resonance) and two-dimensional heteronuclear correlation techniques. Mass spectrometry analyses were carried out at “C.A.C.T.I.—Unidad de Espectrometría de Masas”, University of Vigo, Spain. All commercially available reagents and solvents were used without further purification.

### 3.3. General Procedure for the Synthesis of Eugenol Azo Dyes ***3a***–***e***

The corresponding aniline **1a**–**e** (3.65 × 10^−3^ mol, 2 equiv.) was added to a solution of 1 M HCl (7.5 mL) and 6 M HCl (0.42 mL). After cooling to 0–5 °C, aqueous NaNO_2_ (0.251 g, 1 equiv., in 1 mL of water) was added gradually. The resulting suspension was then stirred for 45 min. The diazonium salt solution was added dropwise to a solution of eugenol (0.300 g, 1.82 × 10^−3^ mol, 1 equiv.) in NaOH (0.120 g, 3.00 × 10^−3^ mol, 1.5 equiv.) and water (1–2 mL) and stirred continuously at room temperature for 10 min. The suspension was filtered off, washed with cold water and diethyl ether, and dried under reduced pressure. The crude products were subjected to column chromatography on silica gel, with dichloromethane/petroleum ether, dichloromethane, and dichloromethane/methanol, as mixtures of increasing polarity as eluent, giving the respective azo dyes **3a** [14], **3b** [16], **3c**, **3d** [14], and **3e**.

#### 3.3.1. 4-Allyl-2-methoxy-6-(phenyldiazenyl)phenol **3a**

Starting from aniline **1a** (0.333 mL, 3.65 × 10^−3^ mol), the crude product was subjected to column chromatography on silica gel, with dichloromethane/petroleum ether as eluent, giving 4-allyl-2-methoxy-6-(phenyldiazenyl)phenol **3a** as an orange solid (0.177 g, 36% yield). *R*_f_ = 0.52 (silica; dichloromethane/petroleum ether 1:1), m.p. = 70.1–72.0 °C. IR (*υ*_max._): 3058, 3002, 2972, 2900, 2844, 2065, 1964, 1636, 1599, 1584, 1316, 1192, 1145, 1098, 1019, 999, 966, 882 cm^−1^. ^1^H NMR (CDCl_3_, 400 MHz): *δ*_H_ 3.43 (2H, d, *J* = 6.8 Hz, C*H*_2_Ph), 3.93 (3H, s, OC*H*_3_), 5.13–5.19 (2H, m, CH=C*H*_2_), 5.98–6.08 (1H, m, C*H*=CH_2_), 6.81 (1H, d, *J* = 1.6 Hz, H-3), 7.42 (1H, d, *J* = 1.6 Hz, H-5), 7.46–7.53 (3H, m, 3 × Ph-*H*), 7.86 (2H, dd, *J* = 7.6 and 1.6 Hz, 2 × Ph-*H*), 13.14 (1H, s, OH) ppm. ^13^C NMR (CDCl_3_, 100.6 MHz): *δ*_C_ 39.45 (*C*H_2_Ph), 56.32 (O*C*H_3_), 115.43 (C-3), 116.09 (CH=*C*H_2_), 122.05 (2 × Ph-*C*), 123.80 (C-5), 129.22 (2 × Ph-*C*), 130.53 (C-6), 131.01 (Ph-*C*), 136.86 (C-4), 137.01 (*C*H=CH_2_), 141.55 (C-1), 148.58 (C-2), 150.21 (C-1 Ph) ppm. HRMS *m*/*z* (ESI): calc for C_16_H_17_N_2_O_2_ [M^+^ + 1] 269.1285; found 269.1275.

#### 3.3.2. 4-Allyl-2-methoxy-6-((3-methoxyphenyl)diazenyl)phenol **3b**

Starting from 3-methoxyaniline **1b** (0.411 mL, 3.65 × 10^−3^ mol), the crude product was subjected to column chromatography on silica gel, with dichloromethane/petroleum ether as eluent, giving 4-allyl-2-methoxy-6-((3-methoxyphenyl)diazenyl)phenol **3b** as an orange solid (0.079 g, 14% yield). *R*_f_ = 0.69 (silica; dichloromethane/petroleum ether 1:1), m.p. = 107.7–109.0 °C. IR (*υ*_max._): 3210, 3075, 2975, 2911, 2834, 1638, 1603, 1482, 1452, 1437, 1413, 1379, 1319, 1287, 1195, 1144, 1110, 1076, 1035, 999, 969, 914, 845 cm^−1^. ^1^H NMR (CDCl_3_, 400 MHz): *δ*_H_ 3.41 (2H, d, *J* = 6.8 Hz, C*H*_2_Ph), 3.87 (3H, s, OC*H*_3_), 3.92 (3H, s, OC*H*_3_), 5.12–5.17 (2H, m, CH=C*H*_2_), 5.99–6.05 (1H, m, C*H*=CH_2_), 6.80 (1H, d, *J* = 1.6 Hz, H-3), 7.01–7.04 (1H, m, *H*-Ph-OCH_3_), 7.39–7.47 (4H, m, H-5 and 4 × *H*-Ph-OCH_3_), 13.10 (1H, s, OH) ppm. ^13^C NMR (CDCl_3_, 100.6 MHz): *δ*_C_ 39.43 (*C*H_2_Ph), 55.33 (O*C*H_3_), 56.30 (O*C*H_3_), 104.62 (C-3), 115.47 (C-5), 116.05 (CH=*C*H_2_), 116.57 (*C*-Ph-OCH_3_), 117.72 (*C*-Ph-OCH_3_), 123.78 (*C*-Ph-OCH_3_), 129.88 (*C*-Ph-OCH_3_), 130.52 (*C*-6), 136.76 (C-4), 137.00 (*C*H=CH_2_), 141.60 (C-1), 148.56 (C-2), 151.38 (C-1 Ph-OCH_3_), 160.38 (C-3 Ph-OCH_3_) ppm. HRMS *m*/*z* (ESI): calc for C_17_H_19_N_2_O_3_ [M^+^ + 1] 299.1390; found 299.1378.

#### 3.3.3. 4-Allyl-2-((3-bromophenyl)diazenyl)-6-methoxyphenol **3c**

Starting from 3-bromoaniline **1c** (0.398 mL, 3.65 × 10^−3^ mol), the crude product was subjected to column chromatography on silica gel, with dichloromethane as eluent, giving 4-allyl-2-((3-bromophenyl)diazenyl)-6-methoxyphenol **3c** as a dark-red solid (0.107 g, 17% yield). *R*_f_ = 0.51 (silica; dichloromethane), m.p. = 147.0–149.0 °C. FTIR (*υ*_max._): 2963, 1639, 1571, 1490, 1463, 1411, 1374, 1272, 1259, 1185, 1142, 1107, 1088, 1001, 964, 939, 911, 883, 844 cm^−1^. ^1^H NMR (CDCl_3_, 400 MHz): *δ*_H_ 3.43 (2H, d, *J* = 6.8 Hz, C*H*_2_Ph), 3.94 (3H, s, OC*H*_3_), 5.14–5.19 (2H, m, CH=C*H*_2_), 5.99–6.06 (1H, m, C*H*=CH_2_), 6.84 (1H, d, *J* = 1.6 Hz, H-3), 7.40 (1H, t, *J* = 8.0 Hz, H-5 Ph-Br), 7.42 (1H, d, *J* = 2.0 Hz, H-5), 7.60 (1H, dq, *J* = 8.0 and 2.0 Hz, H-4 Ph-Br), 7.79 (1H, dq, *J* = 8.0 and 2.0 Hz, H-6 Ph-Br), 8.01 (1H, t, *J* = 2.0 Hz, H-2 Ph-Br) ppm. ^13^C NMR (CDCl_3_, 100.6 MHz): *δ*_C_ 39.52 (*C*H_2_Ph), 56.46 (O*C*H_3_), 116.09 (C-3), 116.32 (CH=*C*H_2_), 122.20 (C-3 Ph-Br), 122.29 (C-6 Ph-Br), 123.38 (C-6), 123.78 (C-2 Ph-Br), 123.98 (C-5), 130.63 (C-5 Ph-Br), 130.94 (C-4), 133.68 (C-4 Ph-Br), 136.96 (*C*H=CH_2_), 141.64 (C-1), 148.68 (C-2), 151.41 (C-1 Ph-Br) ppm. HRMS *m*/*z* (ESI): calc for C_16_H_16_BrN_2_O_2_ [M^+^ + 1] 347.0390; found 347.0376.

#### 3.3.4. 4-Allyl-2-((4-chlorophenyl)diazenyl)-6-methoxyphenol **3d**

Starting from 4-chloroaniline **1d** (0.466 g, 3.65 × 10^−3^ mol), the crude product was subjected to column chromatography on silica gel, with dichloromethane as eluent, giving 4-allyl-2-((4-chlorophenyl)diazenyl)-6-methoxyphenol **3d** as a red solid (0.245 g, 44% yield). *R*_f_ = 0.46 (silica; dichloromethane), m.p. = 136.0–138.0 °C. FTIR (*υ*_max._): 2097, 1640, 1576, 1490, 1422, 1377, 1327, 1264, 1143, 1084, 1001, 965, 912, 841 cm^−1^. ^1^H NMR (CDCl_3_, 400 MHz): *δ*_H_ 3.43 (2H, d, *J* = 6.4 Hz, C*H*_2_Ph), 3.95 (3H, s, OC*H*_3_), 5.12–5.19 (2H, m, CH=C*H*_2_), 5.97–6.07 (1H, m, C*H*=CH_2_), 6.98 (1H, d, *J* = 1.6 Hz, H-5), 7.40 (1H, d, *J* = 2.0 Hz, H-3), 7.50 (2H, d, *J* = 8.8 Hz, H-3 and H-5 Ph-Cl), 7.82 (2H, *J* = 9.2 Hz, H-2 and H-6 Ph-Cl), 12.83 (1H, broad s, OH) ppm. ^13^C NMR (CDCl_3_, 100.6 MHz): *δ*_C_ 39.55 (*C*H_2_Ph), 56.48 (O*C*H_3_), 115.85 (C-3), 116.28 (CH=*C*H_2_), 123.39 (C-2 and C-6 Ph-Cl), 123.86 (C-5), 129.64 (C-3 and C-5 Ph-Cl), 130.88 (C-6), 136.99 (C-4 Ph-Cl), 137.04 (*C*H=CH_2_), 137.07 (C-4), 141.54 (C-1), 148.69 (C-2), 148.89 (C-1 Ph-Cl) ppm. HRMS *m*/*z* (ESI): calc for C_16_H_16_ClN_2_O_2_ [M^+^ + 1] 303.0895; found 303.0882.

#### 3.3.5. 3-((5-Allyl-2-hydroxy-3-methoxyphenyl)diazenyl)-2-bromobenzoic Acid **3e**

Starting from 3-amino-2-bromobenzoic acid **1e** (0.790 g, 3.65 × 10^−3^ mol), the crude product was subjected to column chromatography on silica gel, giving 3-((5-allyl-2-hydroxy-3-methoxyphenyl)diazenyl)-2-bromobenzoic acid **3e** as a dark-red solid (0.298 g, 41% yield). *R*_f_ = 0.64 (silica; dichloromethane/methanol 90:10), m.p. = 174.0–175.0 °C. FTIR (*υ*_max._): 3369, 1695, 1644, 1581, 1558, 1545, 1493, 1392, 1255, 1143, 1100, 1030, 1001, 911 cm^−1^. ^1^H NMR (DMSO-*d_6_*, 400 MHz): *δ*_H_ 3.37 (2H, d, *J* = 6.8 Hz, C*H*_2_Ph), 3.84 (3H, s, OC*H*_3_), 5.06–5.15 (2H, m, CH=C*H*_2_), 5.96–6.03 (1H, m, C*H*=CH_2_), 7.02 (1H, d, *J* = 2.0 Hz, H-4), 7.26 (1H, d, *J* = 2.0 Hz, H-6), 7.53 (1H, t, *J* = 8.0 Hz, H-5 Ph-COOH), 7.66 (1H, d, *J* = 7.6 and 1.6 Hz, H-6 Ph-COOH), 7.85 (1H, d, *J* = 8.0 and 1.6 Hz, H-4 Ph-COOH) ppm. ^13^C NMR (DMSO-*d_6_*, 100.6 MHz): *δ*_C_ 38.81 (*C*H_2_Ph), 56.14 (O*C*H_3_), 116.13 (CH=*C*H_2_), 116.67 (C-4), 116.79 (C-6), 118.37 (C-6 Ph-COOH), 121.22 (C-2 Ph-COOH), 128.33 (C-5 Ph-COOH), 130.72 (C-5), 131.29 (C-4 Ph-COOH), 131.50 (C-1 Ph-COOH), 137.42 (*C*H=CH_2_), 138.03 (C-1), 142.94 (C-2), 148.29 (C-3 Ph-COOH), 148.97 (C-3), 168.04 (*C*OOH) ppm. HRMS *m*/*z* (ESI): calc for C_17_H_16_BrN_2_O_4_ [M^+^ + 1] 391.0288; found 391.0272.

### 3.4. Photophysical Analysis

#### 3.4.1. Preparation of Solutions

Milli-Q-grade ultrapure water and ethanol (spectroscopic grade) were used in all solutions. Stock solutions of several cations, chosen due to their environmental relevance or important biological role, were prepared, at a final concentration of 1 × 10^−2^ M. The salts potassium perchlorate, sodium perchlorate, cesium perchlorate, zinc perchlorate hexahydrate, iron(II) perchlorate hydrate, iron(III) perchlorate hydrate, cadmium perchlorate hexahydrate, nickel perchlorate hexahydrate, copper(II) perchlorate hexahydrate, aluminum perchlorate nonahydrate, lead(II) perchlorate trihydrate, cadmium nitrate tetrahydrate, and cobalt chloride hexahydrate were used for these preparations.

#### 3.4.2. Spectrophotometric Titrations

An analysis of the chemosensor capability of compounds **3a**–**e** was carried out in neutral ethanolic solution (using absolute ethanol), with a 1 × 10^−5^ M compound concentration. A successive addition of each cation was made to 3 mL of each compound solution, and UV/Vis absorption spectra were collected until the absorbance reached a plateau, using a Shimadzu UV-3600 Plus UV-Vis-NIR spectrophotometer and standard quartz cuvettes with a 10.0 mm optical path.

The limit of detection (*LOD*) for each cation was calculated using the slope of the linear range of absorbance versus ion concentration plot and the standard deviation (*s*) of five replicates of absorbance measurements of the analyte-free solution, being calculated through Equation (1) [43]:

(1) LOD=3s/k

where *s* is the standard deviation and *k* is the slope of the calibration curve.

The binding constant was determined using the Benesi–Hildebrand relation (Equation (2)) [44]:

(2) $$\frac{1}{\left(A-A_{0}\right)}=\frac{1}{K_{a}\left(A_{max}-A_{0}\right){\left[ion\right]}^{n}}+\frac{1}{A_{max}-A_{0}}$$

where *A*_0_ is the compound absorbance in the absence of the ion, *A* is the compound absorbance with different ion concentrations, $A_{max}$ is the absorbance of the compound upon complete binding, *n* is the binding stoichiometry, and *K_a_* is the binding constant.

#### 3.4.3. Ab Initio Molecular Quantum Chemistry Calculations

For compound **3a**, the structural and electronic properties were investigated using ab initio molecular quantum chemistry calculations with ORCA software (version 6.0.1) [45], employing a def2-TZVP basis set [46] and using the DFT method with RIJCOSX approximation [47]. def2/J [48] was used as the auxiliary basis. Also, B3LYP functional was employed together with atom-pairwise dispersion correction based on EEQ partial charges (D4) [49] and a conductor-like polarizable continuum model (CPCM) corresponding to ethanol. Excited state calculations were carried out using time-dependent DFT with Tamm–Dancoff approximation (TD-DFT-TDA) [45].

## 4. Conclusions

A set of azo dyes incorporating eugenol into their structures display absorption spectra in the UV and blue regions, with maximum wavelengths at 350 nm and around 430 nm (smaller band), justifying the yellowish color. The dyes without substituents in the phenyl group and with a bromide or methoxy group in the *meta* position relative to the azo group were revealed to be promising as sensors for the detection of lead(II) and copper(II), with low detection limits. Moreover, the titrations could be carried out in neutral ethanol, with no need for organic solvents such as acetonitrile, which is very common in these types of assays using organic compounds as sensors.

Accordingly, this work contributes to the identification of new colorimetric sensors for copper(II) and lead(II) detection by integrating a naturally occurring phenol, namely, eugenol, into their structures, together with substituents in the phenyl ring in the *meta* position relative to the azo group. Some of these structures had been previously synthesized for other purposes, but the present study demonstrates the versatility of these eugenol-derived dyes and broadens their scope of application.

## Figures and Tables

**Table 1:** Maximum absorption wavelengths (λ_max_) and molar absorption coefficients (ε) for compounds **3a**–**e** in neutral ethanolic solution.

Compound	λ_max_ (nm)	ε (M^−1^ cm^−1^)
**3a**	340	1.14 × 10^5^
**3b**	338	8.27 × 10^4^
**3c**	343	1.02 × 10^5^
**3d**	345	9.40 × 10^4^
**3e**	353	7.48 × 10^4^

**Table 2:** Results of TD-DFT calculations of compound **3a** in its *trans* and *cis* conformers, as well as its hydrazone tautomeric form and its complex with Pb^2+^: differences in energy between electronic states and corresponding oscillator strengths (see Section 3.4.3 for calculation details).

Transition	Wavelength (nm)				Oscillator Strength (*f*)			
	*trans*	*cis*	Tautomer	Pb^2+^ Complex	*trans*	*cis*	Tautomer	Pb^2+^ Complex
S_0_ → S_1_	448	476	472	537	7.40 × 10^−4^	4.02 × 10^−2^	4.94 × 10^−2^	2.51 × 10^−3^
S_0_ → S_2_	417	395	445	468	8.15 × 10^−2^	6.80 × 10^−3^	1.29 × 10^−1^	1.30 × 10^−1^
S_0_ → S_3_	328	319	348	396	1.06	2.11 × 10^−2^	9.02 × 10^−1^	1.05 × 10^−2^
S_0_ → S_4_	302	288	313	347	1.76 × 10^−2^	4.96 × 10^−3^	4.05 × 10^−3^	8.59 × 10^−2^
S_0_ → S_5_	296	280	311	335	3.26 × 10^−4^	1.32 × 10^−1^	1.50 × 10^−2^	7.84 × 10^−4^
S_0_ → S_6_	278	275	287	325	5.96 × 10^−2^	5.28 × 10^−3^	9.03 × 10^−4^	5.03 × 10^−2^
S_0_ → S_7_	256	271	278	324	1.97 × 10^−3^	1.19 × 10^−1^	8.14 × 10^−3^	2.99 × 10^−1^
S_0_ → S_8_	253	258	257	318	1.10 × 10^−2^	7.25 × 10^−3^	8.93 × 10^−2^	2.17 × 10^−1^

**Table 3:** LOD values and binding constants for the interaction of compounds **3a**–**c** with lead(II) cations.

Compound	LOD (µM)	*K_a_* (M^−1^)
**3a**	0.19	2.80 × 10^4^
**3b**	0.14	1.33 × 10^4^
**3c**	0.16	1.02 × 10^4^

## Data Availability

Data are contained within the article and Supplementary Materials.

## Supplementary Materials

The following supporting information can be downloaded at https://www.mdpi.com/article/10.3390/molecules30132788/s1. ^1^H and ^13^C NMR spectra and HRMS and FTIR data of compounds **3a**–**e**. Two-dimensional NMR analyses (HSQC and HMBC) of dye **3e**. Cartesian coordinates of compound **3a**.
