# Supplementary material for: New Cation Sensors Based on Eugenol-Derived Azo Dyes
Source: Molecules. 2025 Jun 28;30(13):2788. doi: 10.3390/molecules30132788 (PMC12250961; doi:10.3390/molecules30132788)
Supplement: Supplementary file 1 [file molecules-30-02788-s001.zip › molecules-3669941-supplementary.pdf]

Supplementary Materials

New Cation Sensors Based on Eugenol-Derived Azo Dyes

José R. A. Coelho[1], Ana Rita F. Pacheco[2,3], D. Domingues[1], Ana Rita O. Rodrigues[2,3], Akanni A. Temitope[1], Paulo J. G. Coutinho[2,3], Maria José G. Fernandes[1], Elisabete M. S. Castanheira[2,3], M. Sameiro T. Gonçalves[1,*]

[1] *Centre of Chemistry (CQUM), Department of Chemistry, University of Minho, Campus de Gualtar, 4710-057 Braga, Portugal.*

[2] *Physics Centre of Minho and Porto Universities (CF-UM-UP), Department of Physics, University of Minho, Campus de Gualtar, 4710-057 Braga, Portugal.*

[3] *Associate Laboratory LaPMET, University of Minho, Campus de Gualtar, 4710-057 Braga, Portugal.*

* Correspondence: msameiro@quimica.uminho.pt

^{1}H and ^{13}C NMR Spectra and HRMS and FTIR Data of Compounds 3a-e

The ^{1}H NMR spectra of compounds **3a-e** are shown. These spectra confirm the corresponding structure and purity of each compound. In addition, ^{13}C NMR spectra are also shown. For dye **3e**, two-dimensional NMR analyses (HSQC and HMBC) were performed. The NMR spectra were obtained at an operating frequency of 400.0 MHz for ^{1}H and 100.6 MHz for ^{13}C, using the solvent peak as internal reference at 25 °C. Furthermore, the HRMS and FTIR data of compounds **3a**-**e** are shown. Cartesian coordinates of compound **3a** are also provided.

Table of Contents

Spectral data (^{1}H NMR, ^{13}C NMR, HRMS, and FTIR) for compound **3a** 3

Spectral data (^{1}H NMR, ^{13}C NMR, HRMS, and FTIR) for compound **3b** 6

Spectral data (^{1}H NMR, ^{13}C NMR, HRMS, and FTIR) for compound **3c** 9

Spectral data (^{1}H NMR, ^{13}C NMR, HRMS, and FTIR) for compound **3d** 12

Spectral data (^{1}H NMR, ^{13}C NMR, 2D-NMR, HRMS, and FTIR) for compound **3e** 15

Cartesian coordinates of compound **3a** 18

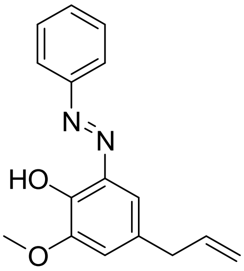

3a

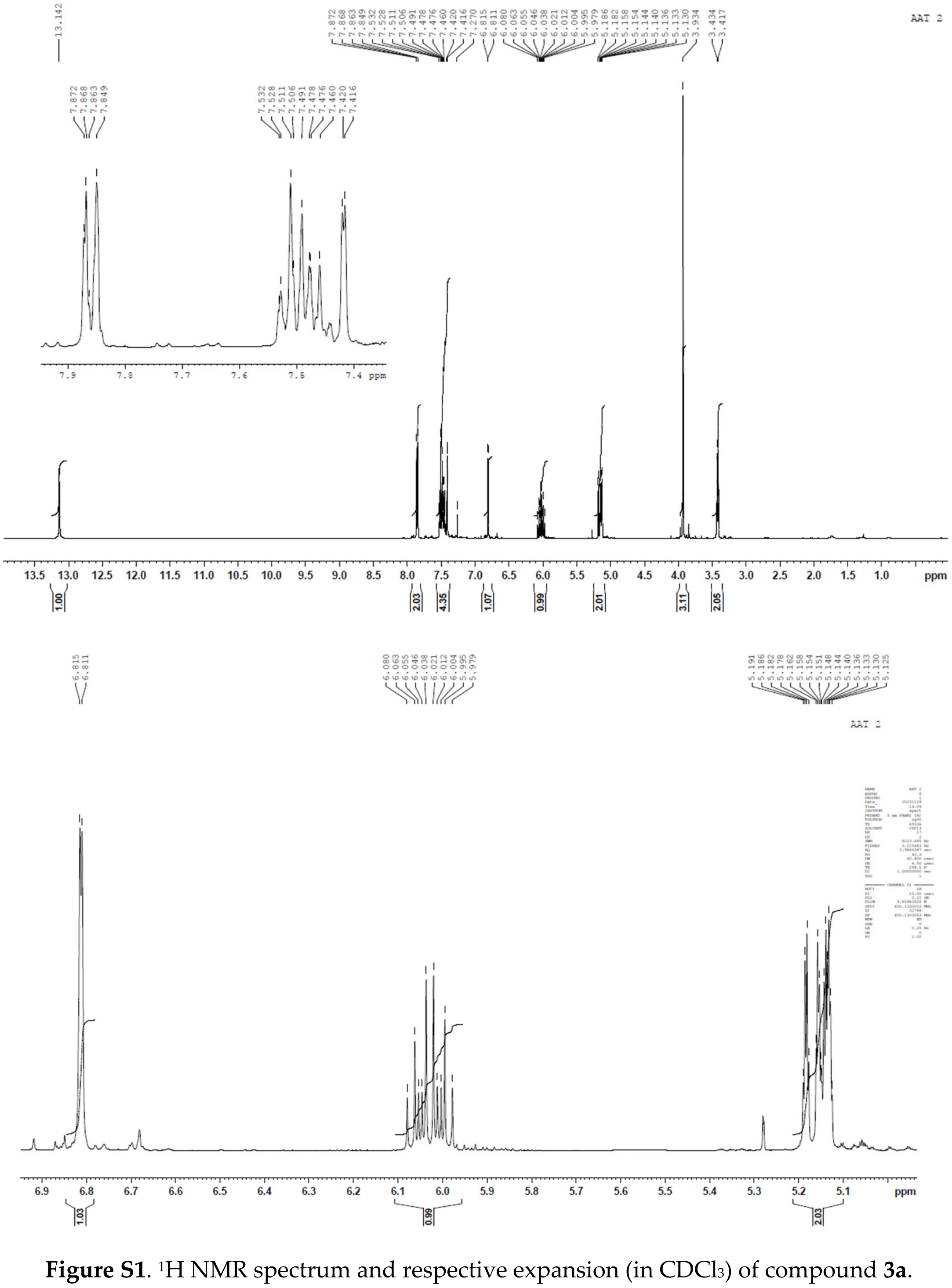

Figure S1. ^{1}H NMR spectrum and respective expansion (in $CDCl_3$) of compound **3a**.

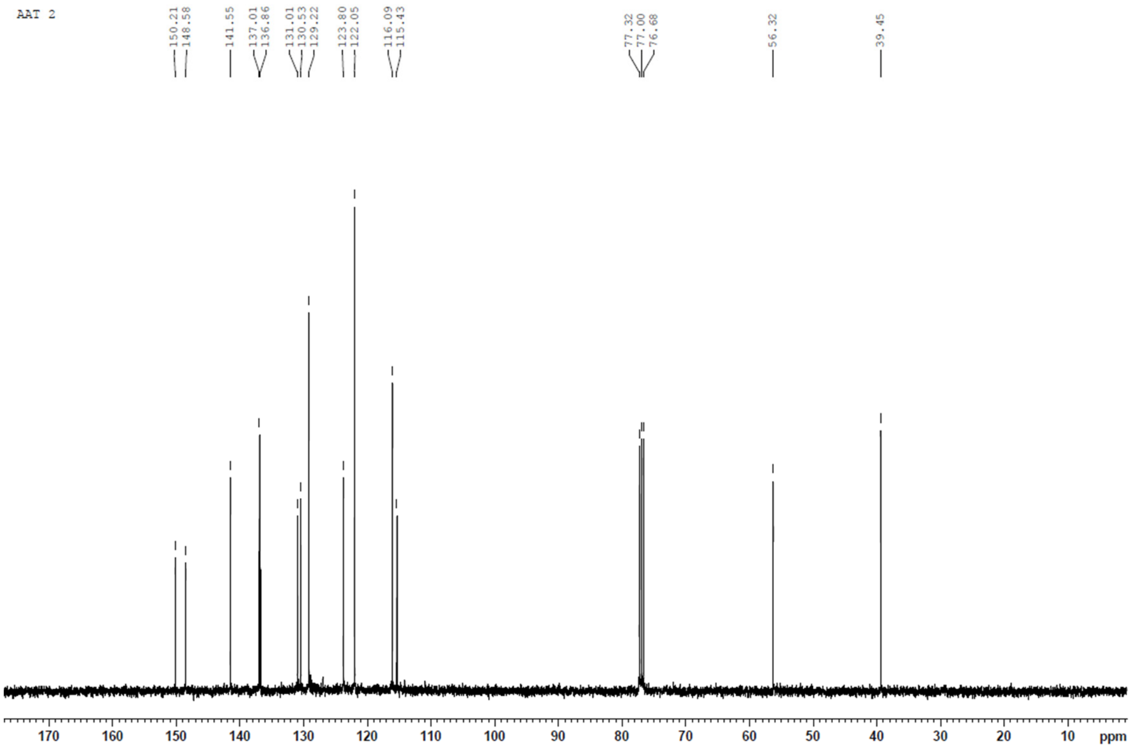

Figure S2. ^{13}C NMR spectrum (in $CDCl_3$) of compound **3a**.

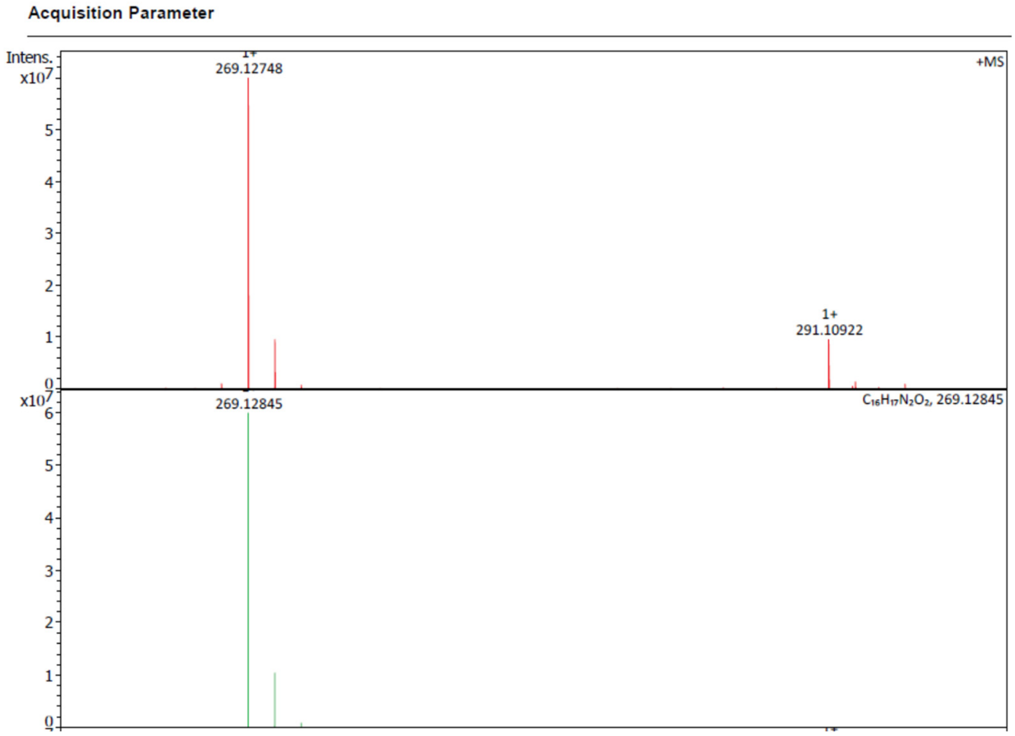

Figure S3. HRMS data of compound **3a**.

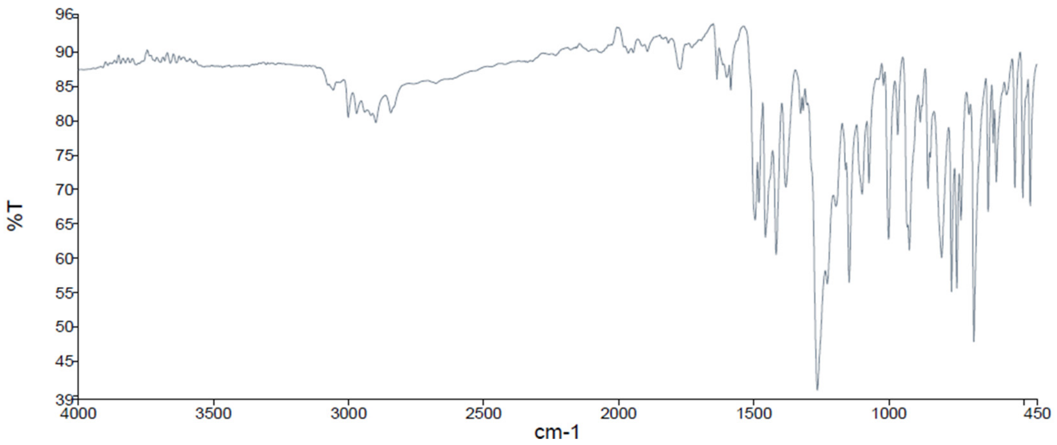

Figure S4. FTIR spectrum of compound **3a**.

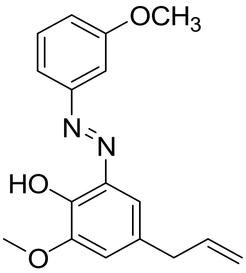

3b

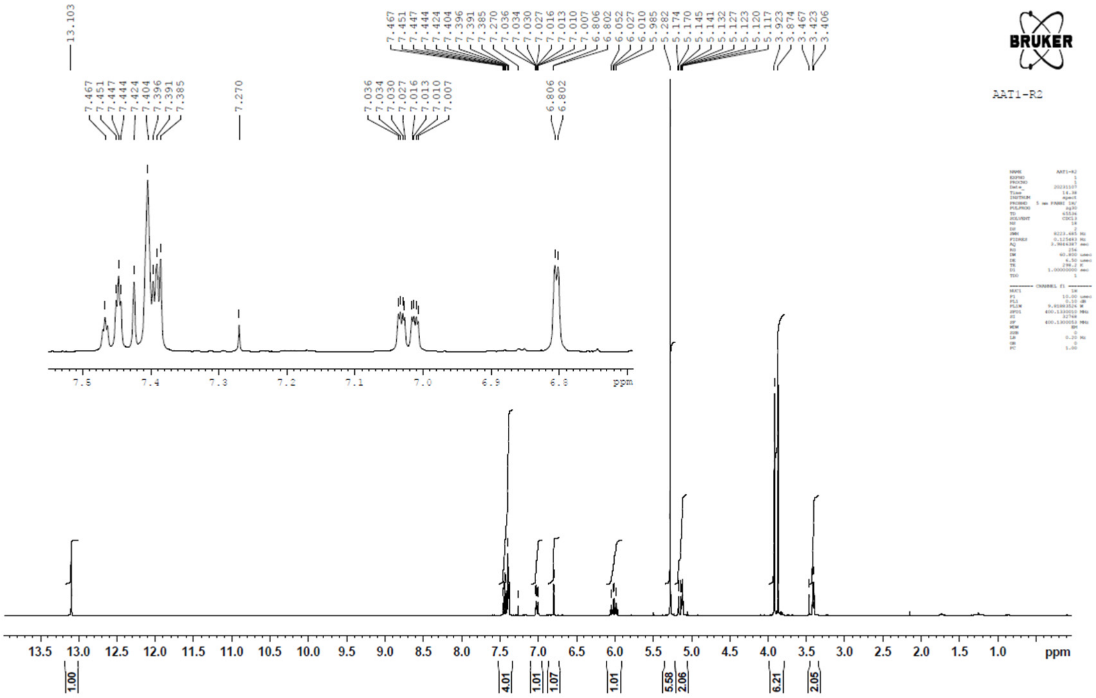

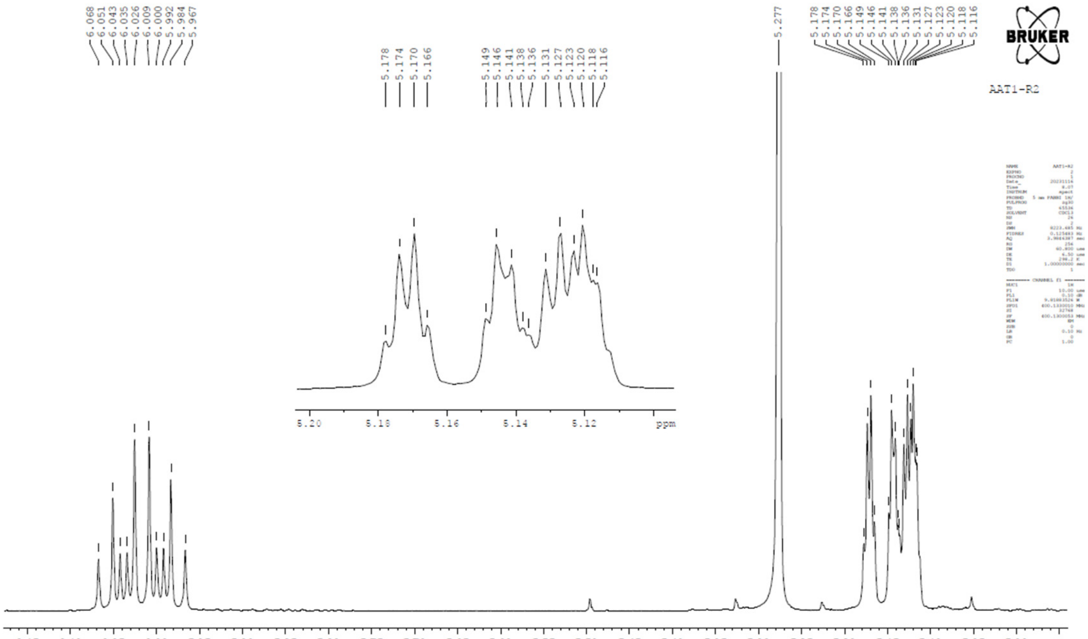

Figure S5. ¹H NMR spectrum and respective expansion (in $CDCl_3$) of compound **3b**.

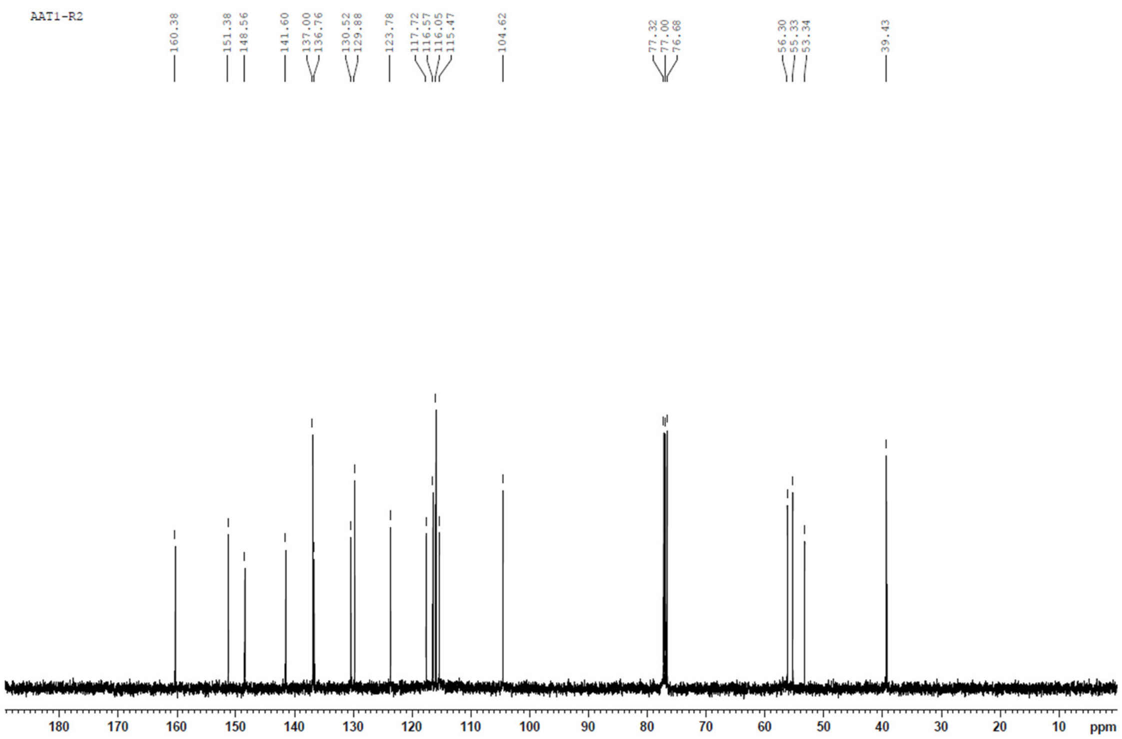

Figure S6. ^{13}C NMR spectrum (in $CDCl_3$) of compound **3b**.

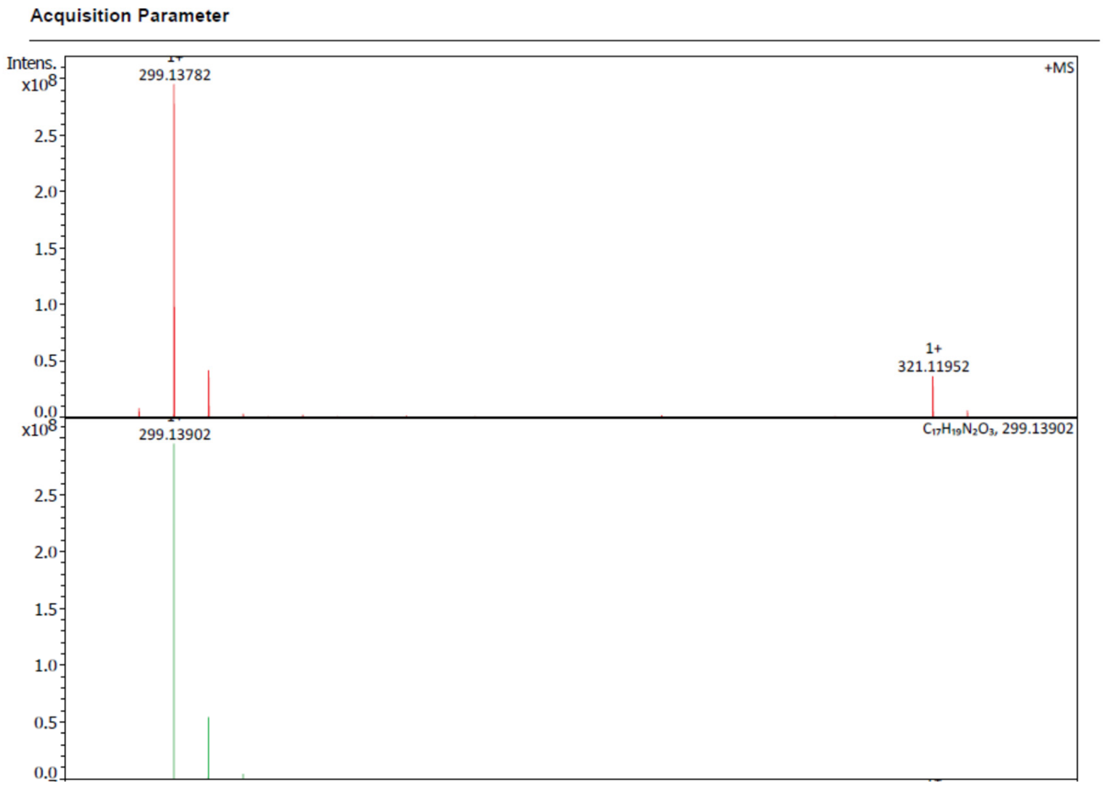

Figure S7. HRMS data of compound **3b**.

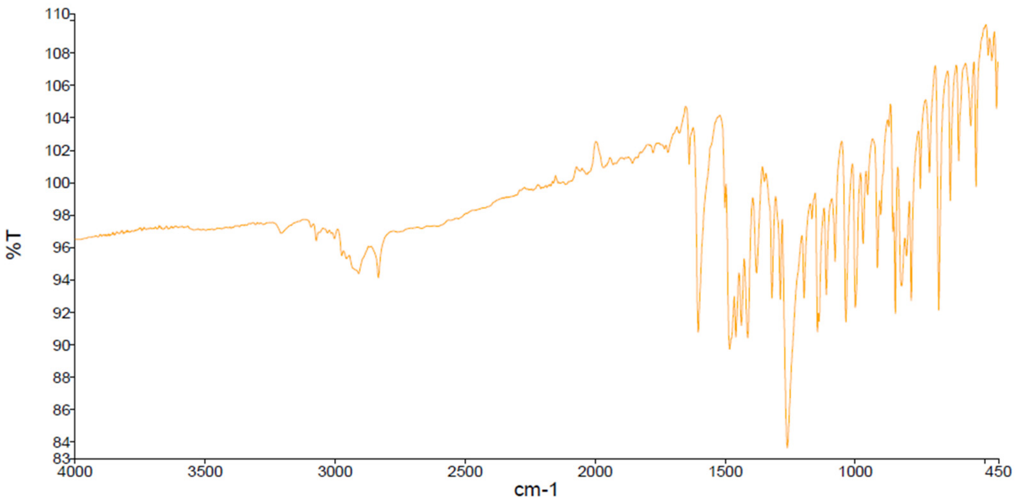

Figure S8. FTIR spectrum of compound **3b**.

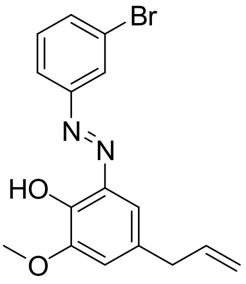

3c

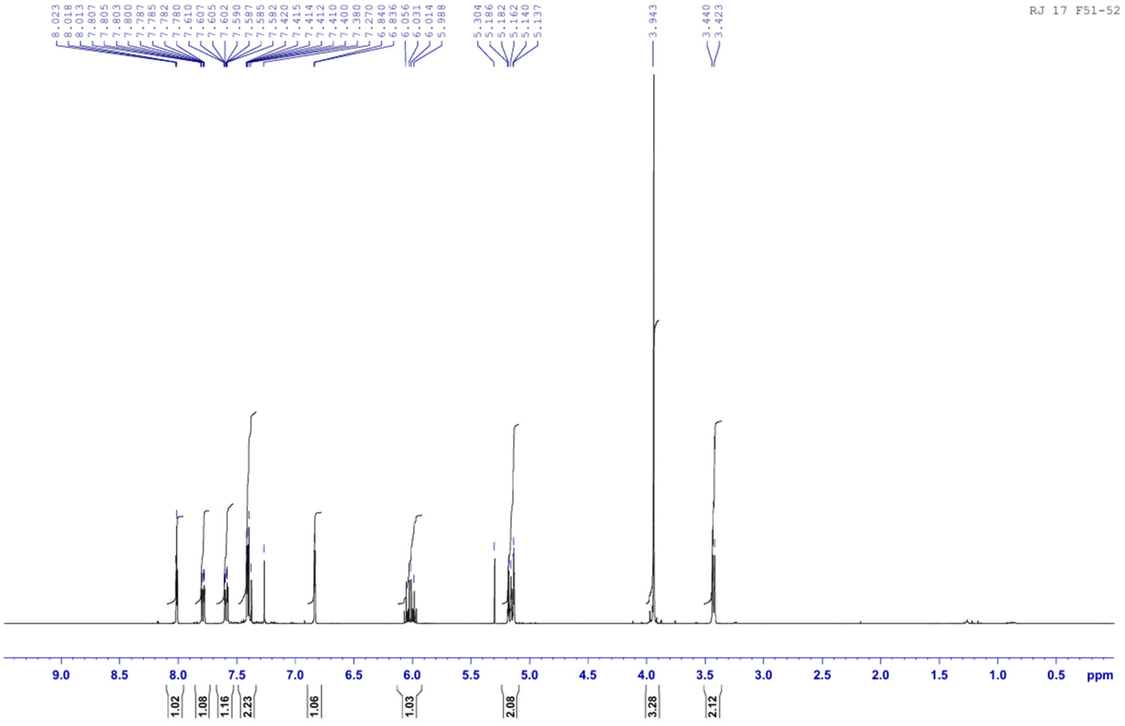

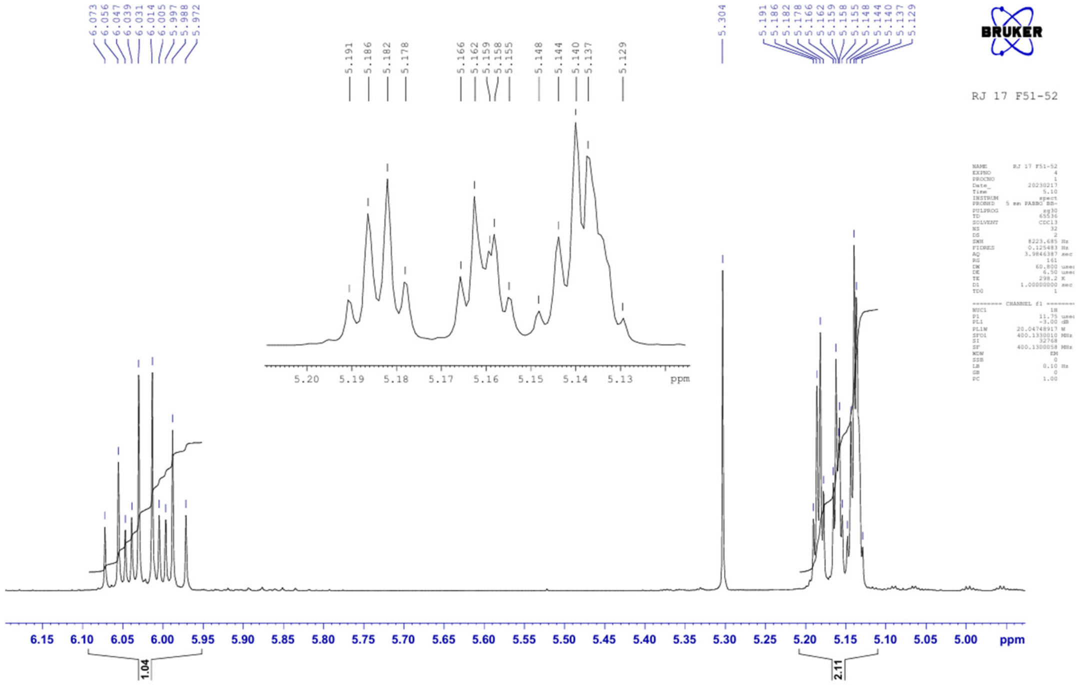

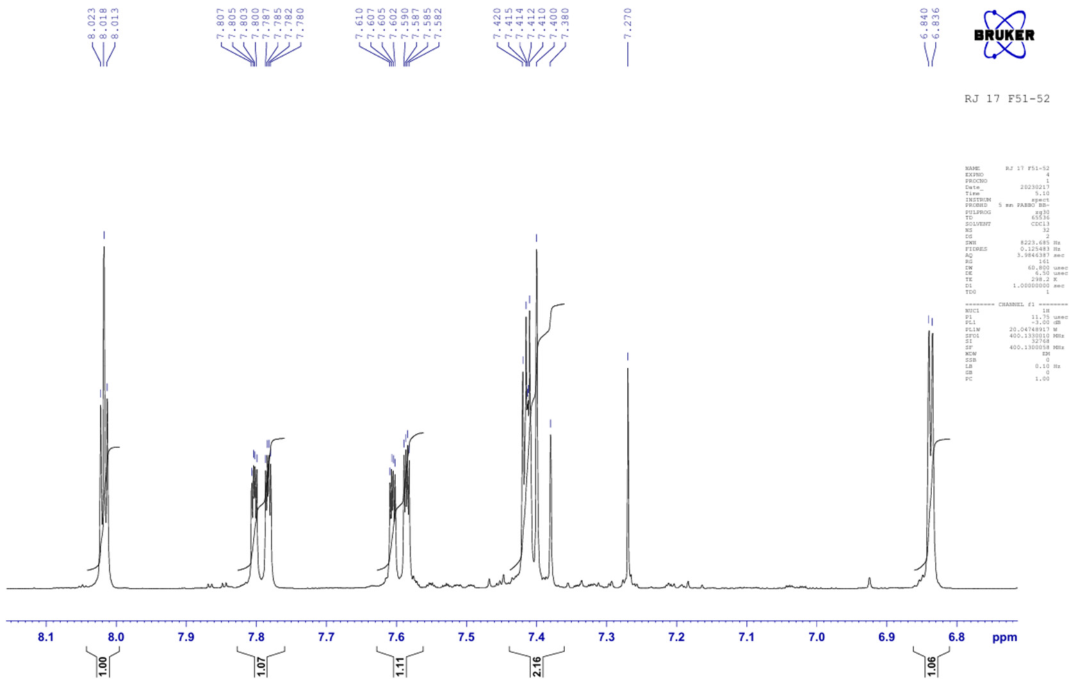

Figure S9. ^{1}H NMR spectrum and respective expansions (in $CDCl_3$) of compound **3c**.

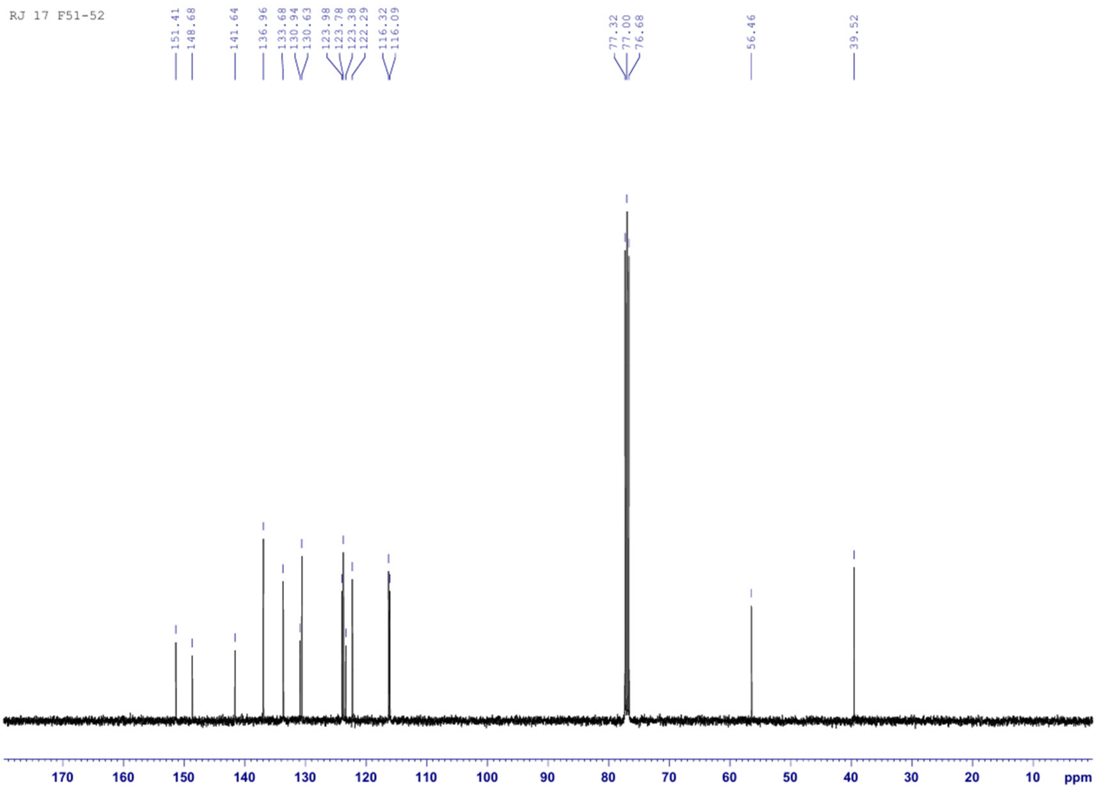

Figure S10. ^{13}C NMR spectrum (in $CDCl_3$) of compound **3c**.

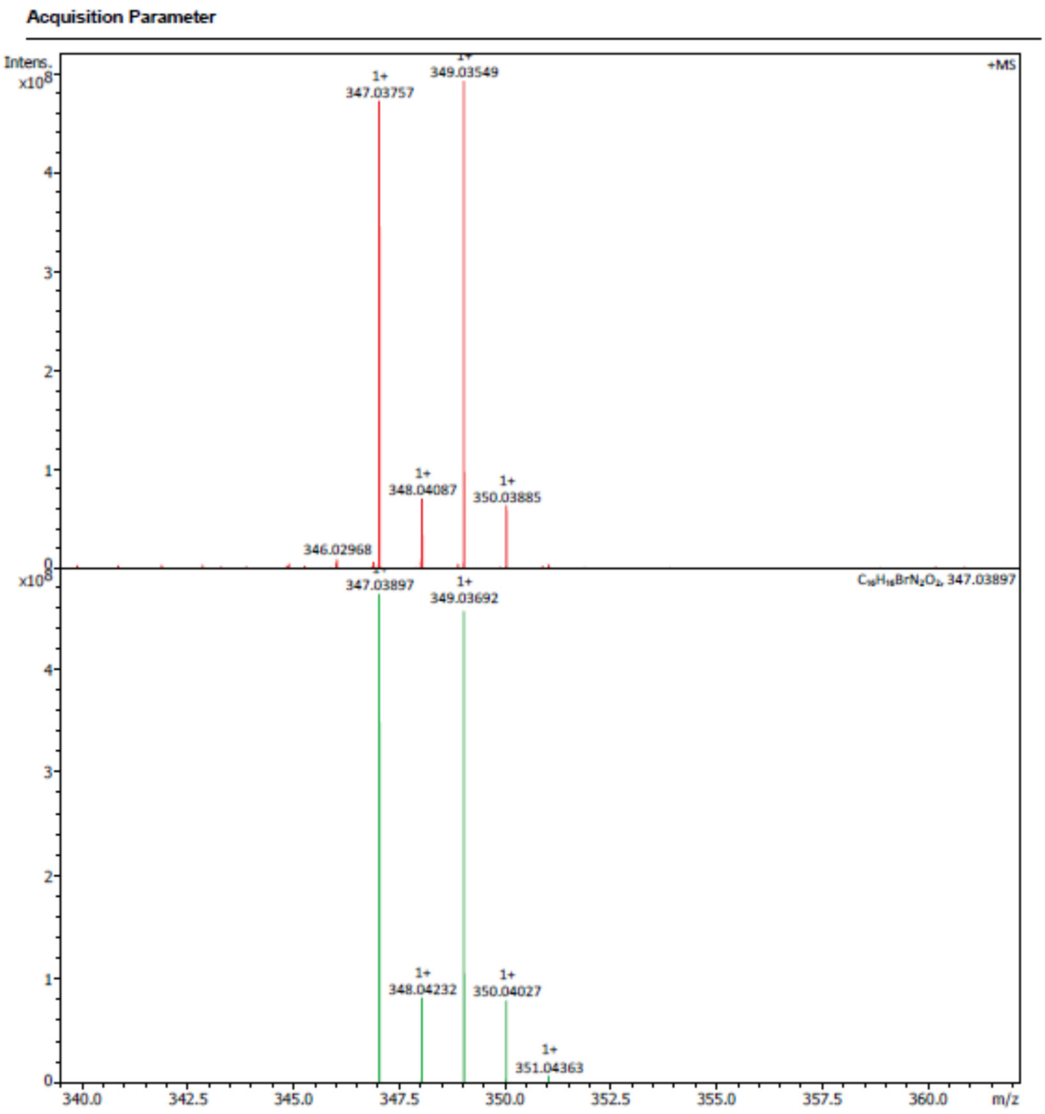

Figure S11. HRMS data of compound **3c**.

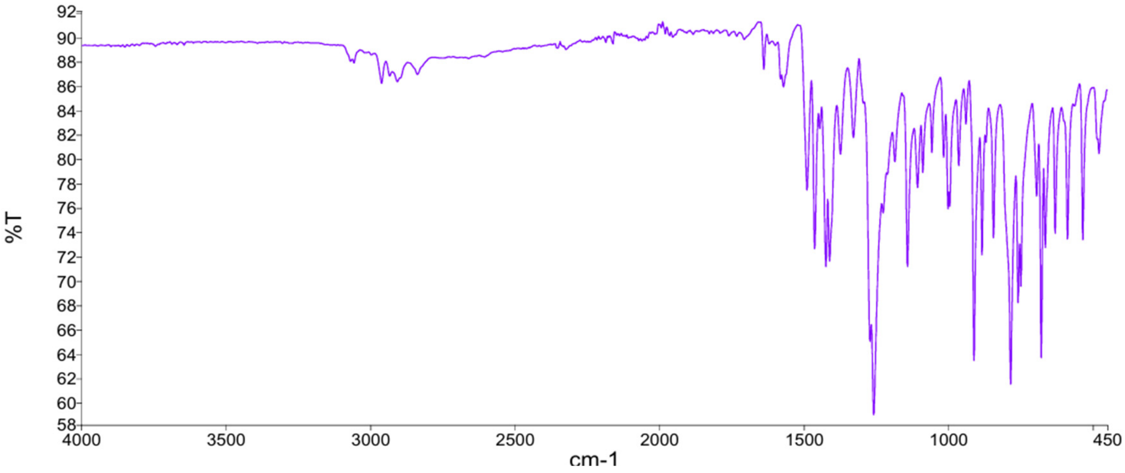

Figure S12. FTIR spectrum of compound **3c**.

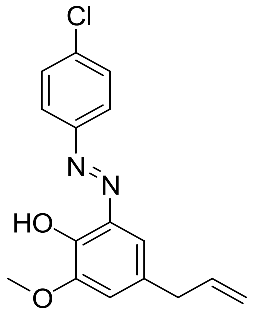
Cl
N
N
HO
O

3d

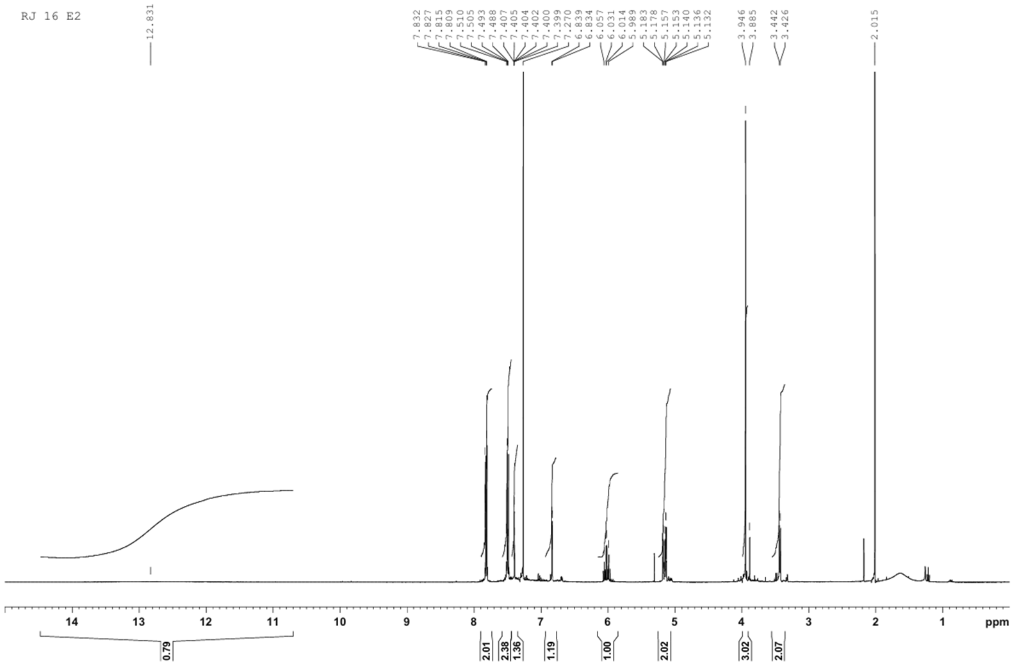
RJ 16 E2
12.831
7.832
7.827
7.815
7.809
7.510
7.505
7.493
7.488
7.407
7.405
7.404
7.402
7.400
7.399
7.270
6.839
6.834
6.057
6.031
6.014
5.989
5.183
5.178
5.157
5.153
5.140
5.136
5.132
3.946
3.885
3.442
3.426
2.015
14 13 12 11 10 9 8 7 6 5 4 3 2 1 ppm
0.79
2.01
2.38
1.36
1.19
1.00
2.02
3.02
2.07

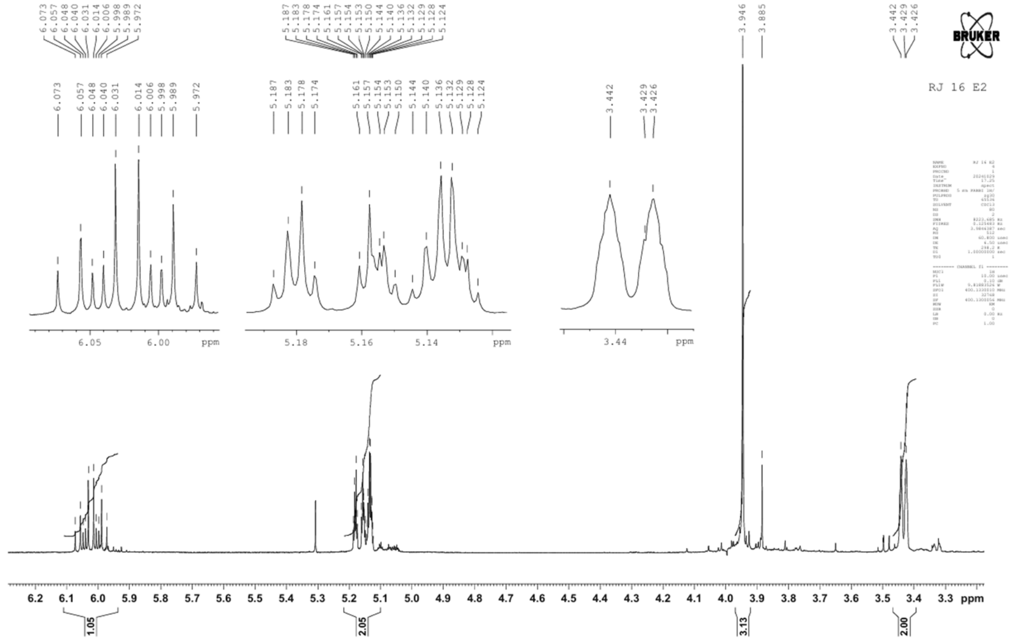
6.073
6.057
6.048
6.040
6.031
6.014
6.006
5.998
5.989
5.972
5.187
5.183
5.178
5.174
5.161
5.157
5.154
5.153
5.150
5.144
5.140
5.136
5.132
5.129
5.128
5.124
3.946
3.885
3.442
3.429
3.426
BRUKER
RJ 16 E2
6.05 6.00 ppm
5.18 5.16 5.14 ppm
3.44 ppm
6.2 6.1 6.0 5.9 5.8 5.7 5.6 5.5 5.4 5.3 5.2 5.1 5.0 4.9 4.8 4.7 4.6 4.5 4.4 4.3 4.2 4.1 4.0 3.9 3.8 3.7 3.6 3.5 3.4 3.3 ppm
1.05
2.05
3.13
2.00

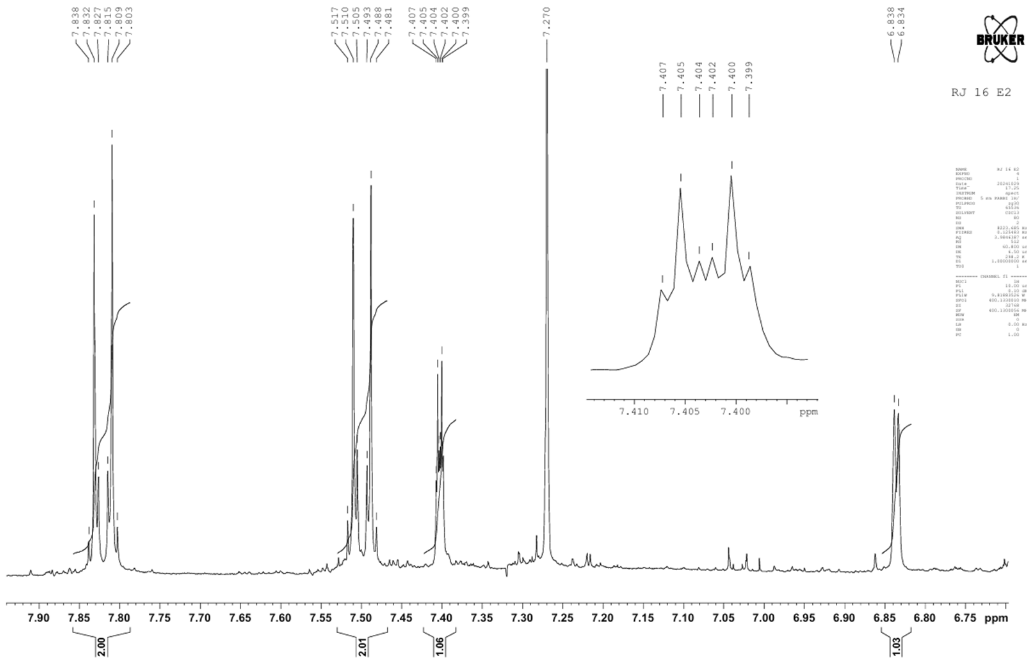

Figure S13. ^{1}H NMR spectrum and respective expansions (in $CDCl_3$) of compound **3d**.

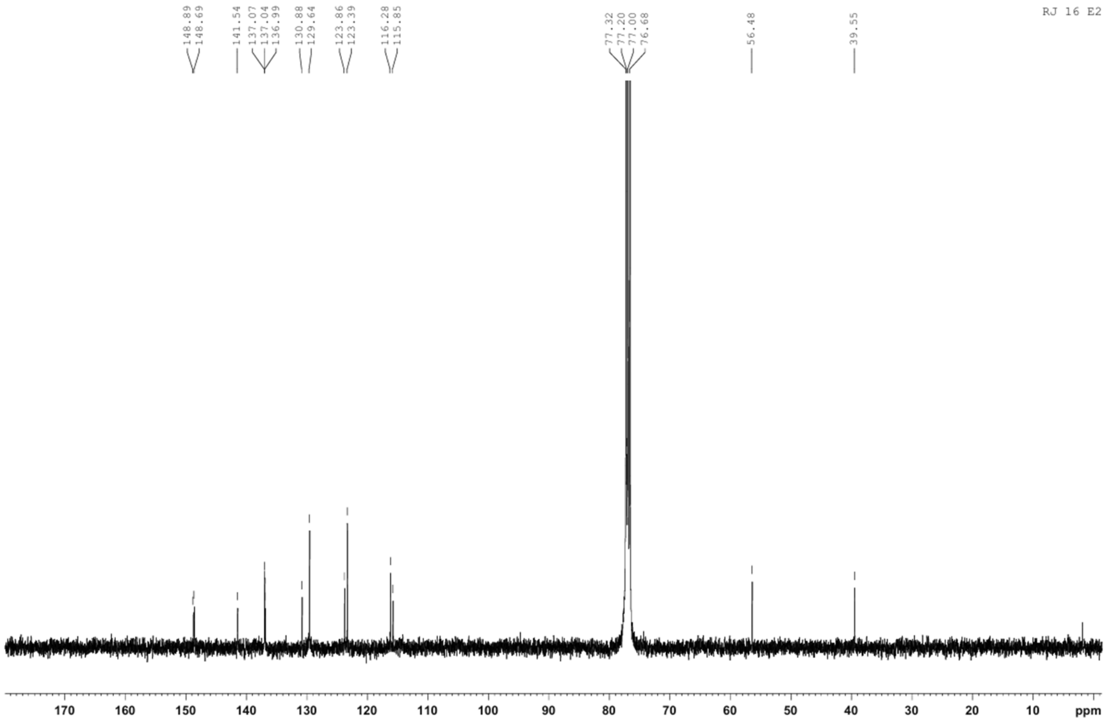

Figure S14. ^{13}C NMR spectrum (in $CDCl_3$) of compound **3d**.

Acquisition Parameter

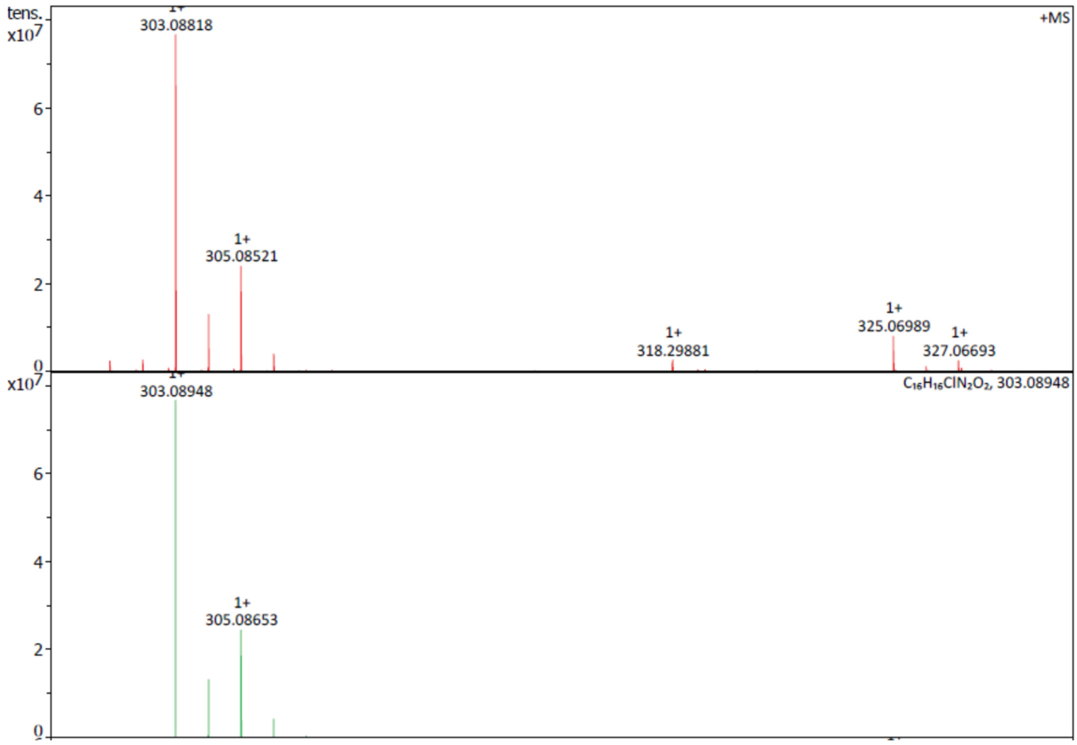

Figure S15. HRMS data of compound **3d**.

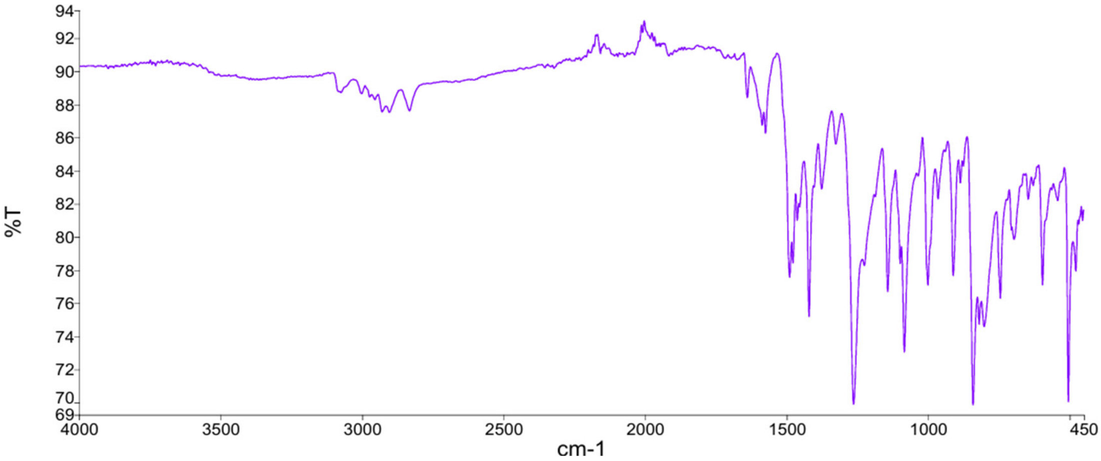

Figure S16. FTIR spectrum of compound **3d**.

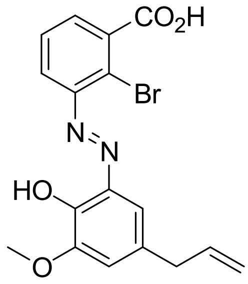

3e

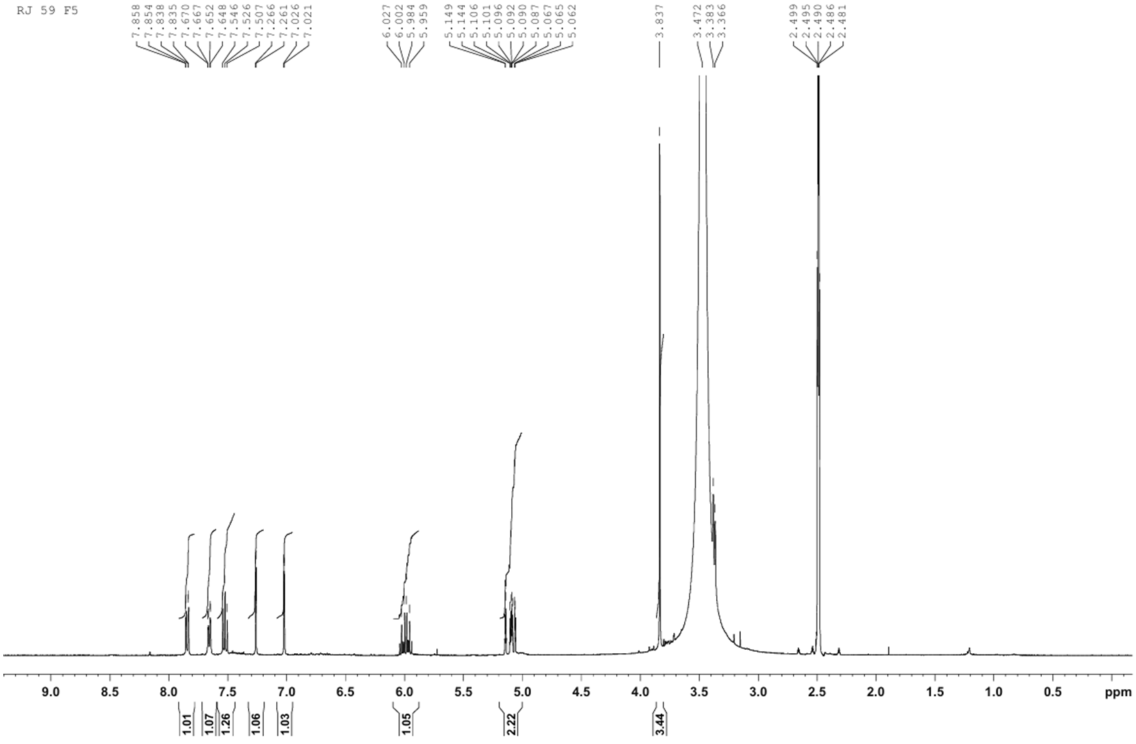

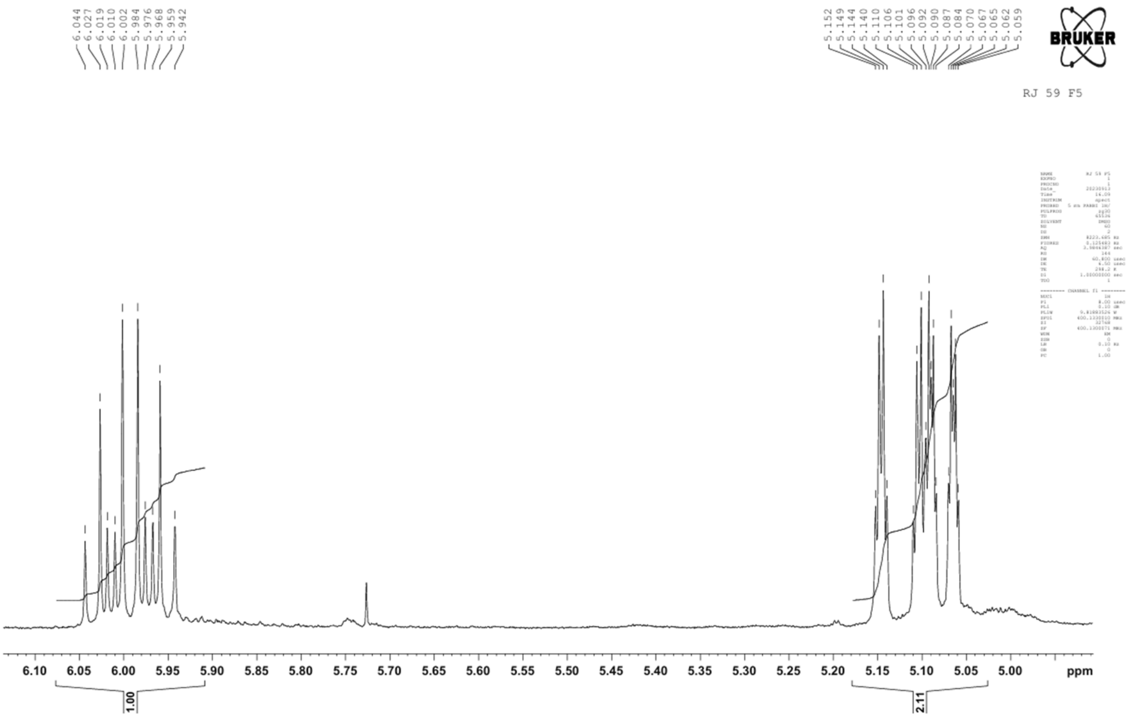

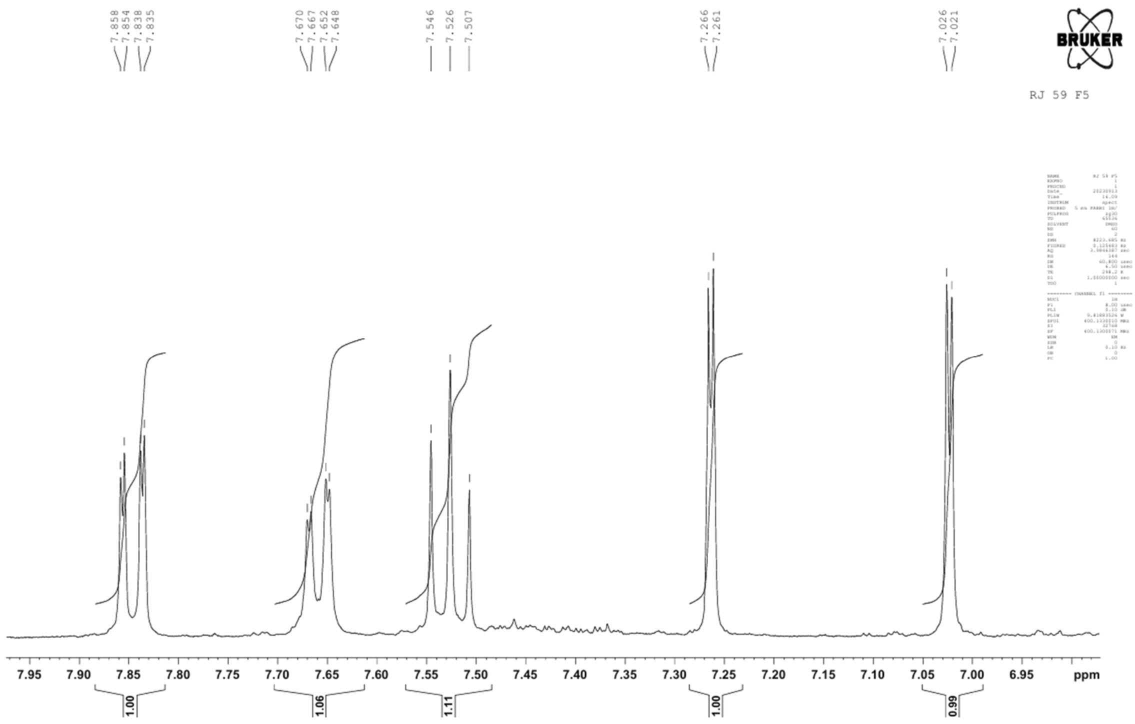

Figure S17. ^{1}H NMR spectrum and respective expansions (in DMSO-d_6) of compound **3e**.

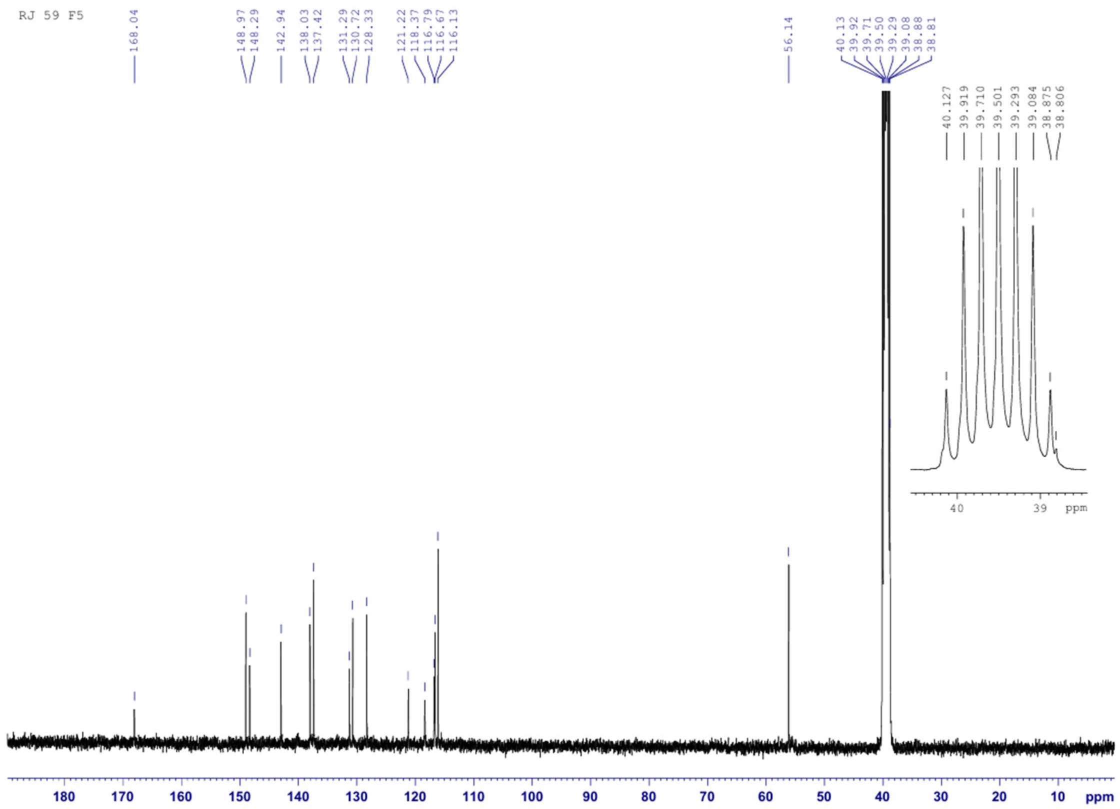

Figure S18. ^{13}C NMR spectrum (in DMSO-d_6) of compound **3e**.

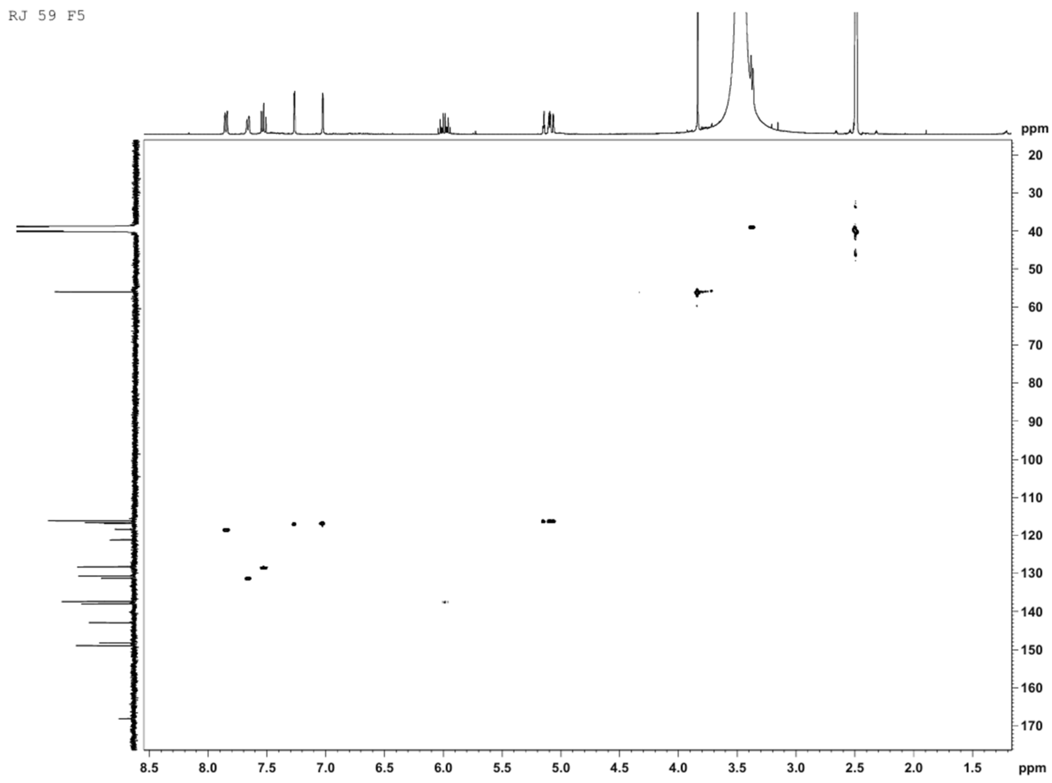

Figure S19. HSQC spectrum of compound **3e**.

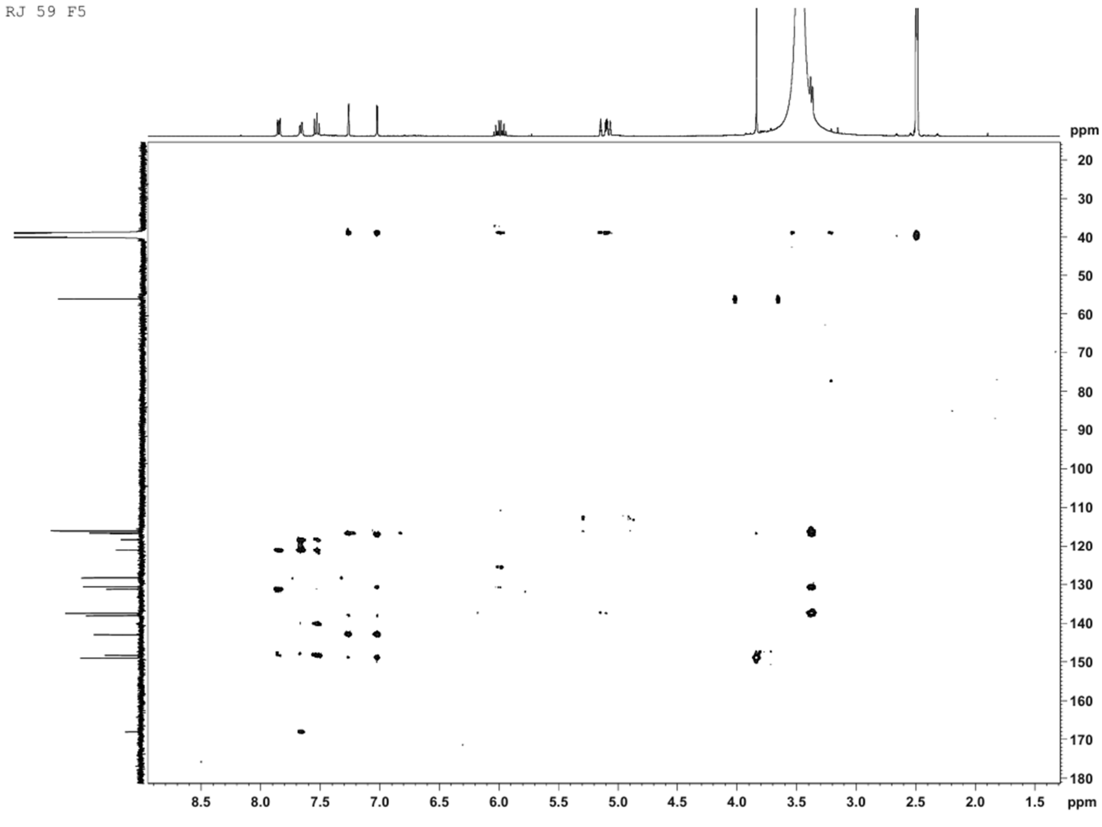

Figure S20. HMBC spectrum of compound **3e**.

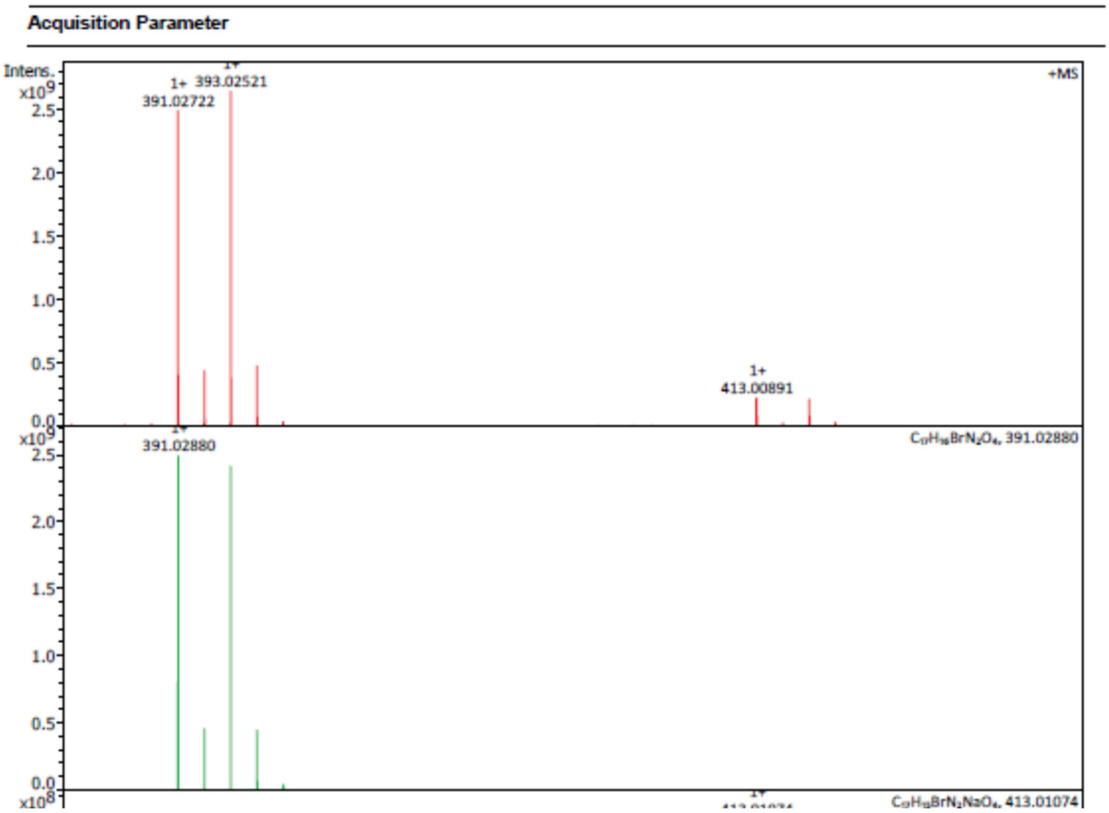

Figure S21. HRMS data of compound **3e**.

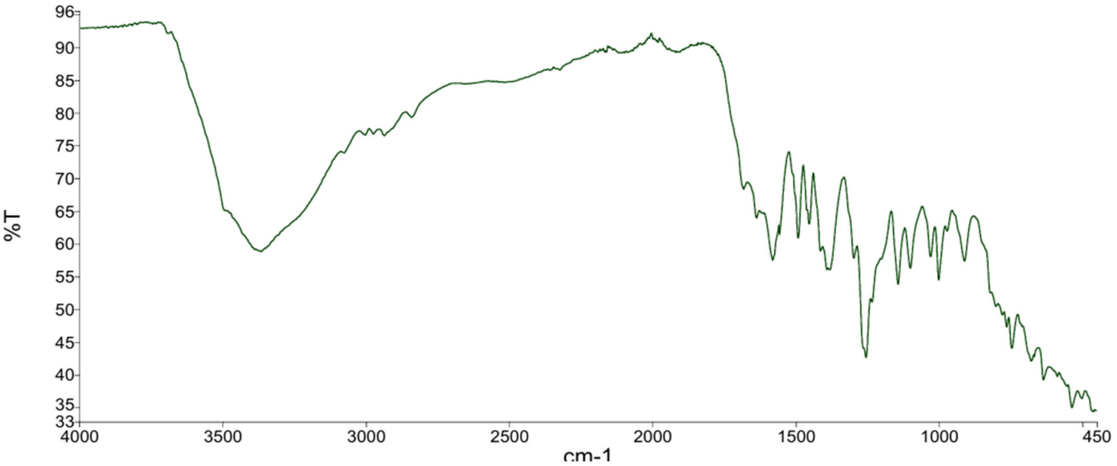

Figure S22. FTIR spectrum of compound **3e**.

Cartesian Coordinates

Table S1. Cartesian coordinates of compound **3a** *(trans)*.

Cartesian coordinates of compound **3a** (***trans***)			
C	-2.25935881771225	-1.65628089677196	-0.15004111570648
C	-0.89258936566861	-1.33509528291266	-0.12945131960552
C	-0.52532753536325	0.02224124274533	0.04783970738113
C	-1.51300795306993	1.00936880278757	0.17927686241194
C	-2.85592887698510	0.69085437717618	0.14471450340456
C	-3.20641107775325	-0.65402040647737	-0.01948586174050
C	-3.92394706362025	1.75390609734375	0.30819370197470
C	-3.66969382969326	2.98242736102009	-0.51431496080515
C	-3.50301403183378	4.20364618635162	-0.02310217575664
N	0.78665215024903	0.48044265289600	0.08653404204906
N	1.69633087253908	-0.38049575559522	-0.04953432894086
C	3.02210291752074	0.09775858495543	-0.00555789467135
C	5.69757139422427	0.85457856942351	0.05348233921838
C	5.35974330348868	-0.48123980586039	-0.12705272515969
C	4.02512313321817	-0.85986313367267	-0.15674674849521
C	4.69428244426411	1.81145116826743	0.20427338862974
C	3.36069171004883	1.44281360087233	0.17606050787112
O	-2.68587944705712	-2.93585524792653	-0.37726854400610
O	0.01655371092406	-2.30350121614730	-0.29338996220258
C	-2.35220091876858	-3.90104510613125	0.62400782599414
H	-1.18220915670502	2.03248464248880	0.29970801613529
H	-4.24846276759642	-0.94865132885392	-0.05696822545602
H	-4.88801682274930	1.32100402109454	0.02339911065574
H	-4.01004161004249	2.03672002939399	1.36242150572883
H	-3.60909145967792	2.82589031035291	-1.58838302432307
H	-3.32084115659333	5.05538827586118	-0.66627029149003
H	-3.54673156420052	4.39578264133990	1.04366070041936
H	6.73789566933465	1.15298533853020	0.07710143585181
H	6.13470120798368	-1.22760040498104	-0.24494883192577
H	3.74240651966273	-1.89529391416067	-0.29725762053302
H	4.95941787062625	2.85187938571231	0.34466674318899
H	2.57328882096362	2.17335951978597	0.29120766647041
H	0.90490355516863	-1.85148520047347	-0.24901749433141
H	-2.81684543414907	-4.83326939314839	0.30866611645816
H	-1.27367130688900	-4.03559950342912	0.70494223815131
H	-2.76219508408812	-3.60108621185710	1.59363471315472

Table S2. Cartesian coordinates of compound **3a** *(cis)*.

Cartesian coordinates of compound **3a** (*cis*)			
C	-2.71856955470258	-1.11939471449947	1.24540937546580
C	-1.34629202034428	-0.88250907726572	1.10027641227725
C	-0.91406867517013	-0.10661010168734	0.01788538145408
C	-1.83469868454719	0.45150221837187	-0.86351890714853
C	-3.19062802866648	0.18396381154270	-0.74492602214174
C	-3.61645462195449	-0.60484645992588	0.32120693879255
C	-4.17026456797003	0.72295946240853	-1.76441271565820
C	-4.09083566130400	-0.01975604450944	-3.06864053819035
C	-3.78554011356813	0.52748288049444	-4.23812069693235
N	0.45927489793195	0.26824622075169	-0.14552059424583
N	1.37436249669545	-0.56313721602733	-0.28202235544667
C	1.14765969584850	-1.96577839485005	-0.43724599977459
C	0.96284548858785	-4.72293528644231	-0.73178612158263
C	1.93872988263718	-4.17839071883373	0.09470003303510
C	2.05012779852937	-2.80144530583781	0.21955998560197
C	0.09999987417979	-3.88493941608484	-1.43238162002746
C	0.17900060574602	-2.50909835868565	-1.28466816589135
O	-3.19112055333891	-1.91708251284486	2.25222361862257
O	-0.51309338242915	-1.41677337155260	2.03402420898512
C	-3.10558489696844	-1.37108548940960	3.57080071701163
H	-1.46128116139628	1.07357550579692	-1.66758543267459
H	-4.66681125124112	-0.82631350297703	0.46435085548652
H	-5.18312421217308	0.63917521063414	-1.35878087964396
H	-3.98276316724924	1.78544280861344	-1.94003882694557
H	-4.28709359101136	-1.08763915044532	-3.01243936305702
H	-3.73496766572851	-0.06154604251796	-5.14522516306698
H	-3.57804828201458	1.58807617540059	-4.33144370514880
H	0.88422944499990	-5.79654729206582	-0.84420873305009
H	2.62521515946998	-4.82467944223600	0.62622100783755
H	2.83031860819047	-2.35522897630692	0.82342389714506
H	-0.64341904398235	-4.30707309616675	-2.09639707772094
H	-0.49207599675498	-1.86442637264684	-1.83192248457784
H	0.40428106861760	-1.19469143340895	1.83971543014259
H	-3.55193270981714	-2.11002356114165	4.23349739971113
H	-2.06970163786886	-1.19479504912138	3.86283258812684
H	-3.67149554123265	-0.43633790652302	3.63395755322976

Table S3. Cartesian coordinates of compound **3a** *(tautomer)*.

Cartesian coordinates of compound **3a** (***tautomer***)			
C	2.26713918115759	1.68051014649669	-0.10631492823024
C	0.85154555147276	1.36003140313730	-0.10515817850538
C	0.52700643726598	-0.06809923907718	-0.03557465243313
C	1.55685058238161	-1.05754013468046	-0.00717176961514
C	2.86781565920531	-0.70326868063762	-0.03990112920357
C	3.19933356928173	0.68717919279033	-0.08474293919047
C	3.99573995629821	-1.71246310234565	0.01094573126610
C	3.61652846947935	-3.09047361984773	-0.43593054620561
C	3.65182950636893	-4.17405687237098	0.32846216279238
N	-0.72534786377088	-0.52487125486428	-0.01112219431151
N	-1.70156478877222	0.32185612339355	-0.05903416992397
C	-3.03532517289747	-0.09164712183125	-0.02243622644675
C	-5.72139896272095	-0.80329744104025	0.04155386394075
C	-5.35821740976063	0.53553716273792	-0.05379683751562
C	-4.01986254561664	0.89585025779169	-0.08617987990166
C	-4.73261577010706	-1.78195175822839	0.10452124772634
C	-3.39087828960569	-1.43792118890709	0.07359349078430
O	2.68320364532407	2.97204291789278	-0.23517989425023
O	-0.04093018919943	2.24080748492135	-0.17327678792247
C	2.23570272840798	3.90400884106009	0.75548610828836
H	1.24803315057877	-2.09310495467674	0.04006095846392
H	4.24237130887137	0.98287650516904	-0.11358925951871
H	4.81067084112124	-1.34375791373354	-0.62288272572837
H	4.40146856227078	-1.75554559399344	1.02803211402251
H	3.28540589460308	-3.17755363434700	-1.46778017421231
H	3.37237241317765	-5.14815404303279	-0.05291721527115
H	3.96610497521029	-4.12615741177557	1.36558056455452
H	-6.76631098853225	-1.08326645196299	0.06689577186970
H	-6.11899548228706	1.30375504955419	-0.10350780401309
H	-3.73033088316604	1.93691473931643	-0.16150121470720
H	-5.01062885831713	-2.82560895135489	0.17907356439974
H	-2.61633182655191	-2.18890457235722	0.12216614907345
H	-1.43174080950299	1.32321598716439	-0.12287211602149
H	2.73963629157115	4.84144119434389	0.52822346959864
H	1.15634407003562	4.03742122749370	0.71456227740276
H	2.53297704672487	3.56729570780170	1.75391316894460

Table S4. Cartesian coordinates of compound **3a** *(Pb^{2+} complex)*.

Cartesian coordinates of compound **3a** (*Pb^{2+} complex*)			
C	-2.16548728263218	-1.52180895616374	0.11712940790952
C	-0.74273094096994	-1.31645206660816	0.08092413423605
C	-0.30258143705102	0.02889096716372	0.33646714851311
C	-1.23222647324481	1.04841722565096	0.62926041306094
C	-2.58615621929945	0.81728453836678	0.65533212284070
C	-3.03473942497160	-0.48916278021406	0.39452612547705
C	-3.57477069568126	1.93245051786519	0.91343055518082
C	-4.00575992082957	2.61846158160838	-0.35181940210868
C	-3.89264146362384	3.91848047637503	-0.59746823204499
N	1.01076320829471	0.47235060458250	0.34192321408602
N	1.92660798504936	-0.29646287605778	-0.05206553596352
C	3.21870007306035	0.27899863604489	-0.02382382277771
C	5.83996647871299	1.23828265336086	-0.05129772415639
C	5.59106507810707	-0.12332712004569	-0.18620276916622
C	4.28871540100560	-0.60704269914097	-0.17981725993607
C	4.77055476650633	2.12152676247803	0.09338433645081
C	3.46771915531975	1.65097246463685	0.10901440948688
O	-2.73275551403440	-2.72196692479048	-0.20662789071666
O	0.03591773867922	-2.31004017568661	-0.14397829176737
C	-2.13144330656612	-3.94976978550078	0.22741479575975
H	-0.82912502648382	2.03637024445074	0.81872449780748
H	-4.09560179037249	-0.71706758917050	0.40048898937714
H	-4.45916803362543	1.51715073750185	1.41147191343030
H	-3.14360767514143	2.67105282321230	1.59466842519983
H	-4.41575988184211	1.96579497785368	-1.11901658949304
H	-4.20311657927140	4.34973222294469	-1.54157490828659
H	-3.47043971268654	4.59778618580171	0.13596105178741
H	6.85691333857262	1.61272426540837	-0.06417824804917
H	6.41581712184728	-0.81694194071955	-0.30077396598368
H	4.07412144623599	-1.66736506181459	-0.28164036107013
H	4.95864772679700	3.18517540898054	0.18918892693831
H	2.62797103331918	2.32289447529601	0.21750166807910
H	-2.95579991535376	-4.66502011323162	0.28040609608104
H	-1.37203593513335	-4.29598893259685	-0.47215899706301
H	-1.68697262492429	-3.83806265138971	1.21866701458152
Pb	1.48790689210678	-2.83771482984868	-1.88850423251485
O	2.91634079066450	-3.59560452418919	-0.42825481481691
O	0.54795555400699	-4.79453053463194	-2.04540642175016

H	0.97098300110107	-5.32456295203923	-1.35811625347802
H	2.55675306435199	-3.34700525574291	0.43214047485940
